# Bone metastasis classification using whole body images from prostate cancer patients based on convolutional neural networks application

**DOI:** 10.1371/journal.pone.0237213

**Published:** 2020-08-14

**Authors:** Nikolaos Papandrianos, Elpiniki Papageorgiou, Athanasios Anagnostis, Konstantinos Papageorgiou

**Affiliations:** 1 General Department, University of Thessaly, Lamia, Greece; 2 Faculty of Technology, Dept. of Energy Systems, University of Thessaly, Geopolis Campus, Larisa, Greece; 3 Institute for Bio-economy and Agri-technology, Center for Research and Technology Hellas, Greece; 4 Department of Computer Science and Telecommunications, University of Thessaly, Lamia, Greece; Korea National University of Transportation, REPUBLIC OF KOREA

## Abstract

Bone metastasis is one of the most frequent diseases in prostate cancer; scintigraphy imaging is particularly important for the clinical diagnosis of bone metastasis. Up to date, minimal research has been conducted regarding the application of machine learning with emphasis on modern efficient convolutional neural networks (CNNs) algorithms, for the diagnosis of prostate cancer metastasis from bone scintigraphy images. The advantageous and outstanding capabilities of deep learning, machine learning's groundbreaking technological advancement, have not yet been fully investigated regarding their application in computer-aided diagnosis systems in the field of medical image analysis, such as the problem of bone metastasis classification in whole-body scans. In particular, CNNs are gaining great attention due to their ability to recognize complex visual patterns, in the same way as human perception operates. Considering all these new enhancements in the field of deep learning, a set of simpler, faster and more accurate CNN architectures, designed for classification of metastatic prostate cancer in bones, is explored. This research study has a two-fold goal: to create and also demonstrate a set of simple but robust CNN models for automatic classification of whole-body scans in two categories, malignant (bone metastasis) or healthy, using solely the scans at the input level. Through a meticulous exploration of CNN hyper-parameter selection and fine-tuning, the best architecture is selected with respect to classification accuracy. Thus a CNN model with improved classification capabilities for bone metastasis diagnosis is produced, using bone scans from prostate cancer patients. The achieved classification testing accuracy is 97.38%, whereas the average sensitivity is approximately 95.8%. Finally, the best-performing CNN method is compared to other popular and well-known CNN architectures used for medical imaging, like VGG16, ResNet50, GoogleNet and MobileNet. The classification results show that the proposed CNN-based approach outperforms the popular CNN methods in nuclear medicine for metastatic prostate cancer diagnosis in bones.

## 1. Introduction

Most common tumors, such as those of the breast, lung and prostate, frequently metastasize to the bone tissue, and so, the skeleton seems to be a site with the most significant tumor burden in cancer patients with advanced disease. Statistical analysis results have shown that 65% of all bone metastases originate from the breast in women and from the prostate in men. The remaining 35% emanates from thyroid, lung and kidney cancers [1]. In the case of metastatic prostate cancer, diagnosis has a significant impact on the quality of patient’s life [2]. In most men, the metastatic prostate cancer mainly sites on the bones of the axial skeleton, causing severe lesions that can cause pain, debility and/or functional impairment [3]. As this type of cancer has great avidity for bone and could cause painful and untreatable effects, an early diagnosis is crucial for the patient. Reviews on clinical evidences and diagnostic assessments of bone metastases in men with prostate cancer can be found in [4].

The implementation of a properly selected diagnostic imaging can reveal the number of metastatic foci in the skeletal system [1, 5]. Rapid diagnosis of bone metastases can be achieved using modern imaging techniques such as scintigraphy, Positron Emission Tomography (PET) and whole-body Magnetic Resonance Imaging (MRI).

The primary imaging method in the diagnosis of metastases, that offers the highest sensitivity among all imaging methods (95%), is Bone Scintigraphy (BS) [6–8]. Through the depiction of the entire skeleton in one medical examination, nuclear doctor is able to detect bone abnormalities in areas where intensive radionuclide activity is present. However, low specificity seems to be the main drawback of this method, as it cannot tell whether the causes of bone turnovers are different than those of metastatic origin (leukaemia, healing fracture, etc.). At the same time, PET has been recognized as an efficient method for detecting cancer cells, based on recent technological advancements in medical imaging. PET and Computed Tomography (CT) combination can produce high-resolution images [9–11].

Although PET and PET/CT are the most efficient screening techniques for bone metastasis, BS remains the most common imaging procedure in nuclear medicine [9, 12], [13]. As reported in European Association of Nuclear Medicine (EANM) guidelines [13], BS is particularly important for clinical diagnosis of metastatic cancer, both in men and women. At present, when other imaging or examination methods are unable to provide a reliable diagnosis, BS imaging becomes the proper modality for making a final diagnosis of bone metastasis [14].

To address the considerable problem of bone metastasis diagnosis, artificial intelligent methods for medical image analysis, implemented with deep learning algorithms, have been adequately investigated. In this direction, a recent survey reveals the entire penetration of deep learning techniques into the field of medical image analysis; detection, segmentation, classification, retrieval, image generation and enhancement, registration and successful application of deep learning to medical imaging tasks are thoroughly examined [15–18].

Implementation of deep learning in medical imaging is mainly conducted by Convolutional Neural Networks (CNNs) [16, 19, 20], a relatively new and powerful way to learn useful representations of images and other structured data. Before the application of CNNs, these features typically had to be created by less powerful machine learning models or even hand-crafted. With the introduction of CNNs, such features could be learned directly from the provided data, since they include certain preferences in their structure that make them powerful deep learning models for image analysis [15, 20, 21]. Typical CNNs have a similar structure with Artificial Neural Networks (ANN) and consist of one or more filters (i.e., convolutional layers), followed by aggregation/pooling layers in order to extract features for classification tasks [22]. Gradient descent and backpropagation are both used as learning algorithms, the same way they are used in a standard ANN. Their main difference lies in the fact that CNNs have layers of convolutions along with pooling layers in the beginning of their architecture. The final outputs are computed via fully connected layers, located at the end of the network architecture [18].

In recent years, CNNs have gained wider recognition in medical image analysis domain, as well as in vision systems [15–18, 20]. Due to their enormous popularity, several applications of CNNs were investigated in the field of medical image analysis. Two recent review studies, [15] and [18], gather all the important and most interesting applications of deep learning.

The application of CNNs in medical imaging ranges from plain radiograph, CT, MRI and microscopy images to clinical photos (dermatology), capsule endoscopy and visual recognition [23–29]. In addition, a CNN was investigated in [30] that regards automatic detection of tuberculosis on chest radiographs, while in [31], a brain tumor segmentation in magnetic resonance images was made possible with the use of a CNN. More examples of CNNs’ successful application in medical domain include automated cardiac diagnosis [32], detection of lesions and prediction of treatment response by PET [33, 34], as well as dynamic contrast agent-enhanced computed tomography, where CNN showed high diagnostic performance in the differentiation of liver masses [35]. Furthermore, CNNs have shown outstanding performance in radiology and molecular imaging [25].

Some models with major impact in the context of deep learning and medical image processing were introduced in several research articles: the U-net model for biomedical data semantic segmentation [36], the GoogLeNet model introducing the inception module [37], the ResNet model introducing the residual learning building block for extremely deep convolutional networks [38] and also, Deeplab, which deals with the inclusion of many convolutional layers atrous for semantic segmentation of images in deep convolutional neural networks [39].

In medical image analysis, the most widely used CNN methods are the following: (i) **AlexNet** (2012) [40]: This network has a quite deep architecture, similar to GoogLeNet, by Yann LeCun et. al [41], incorporates more filters per layer and includes stacked convolutional layers. It attaches ReLU activations after every convolutional and fully-connected layer. (ii) **ZFNet** (2013) [42]: Being a rather slight modification of AlexNet, this network won the 2013 ILSVRC competition. **(iii) VGGNet16** (2014) [43, 44]: It consists of 16 convolutional layers, having a very uniform architecture, similar to AlexNet. (iv) **GoogleNet** [37]: It is a convolutional neural network with a standard stacked convolutional layer, having one or more fully connected layers, called inception modules, able to extract various levels of features on the same time. (v) **ResNet** (2015) [38]: It is another efficient CNN architecture that introduced the “identity shortcut connection”, to solve the notorious problem of the vanishing gradients of the deep networks. (vi) **DenseNet** (2017) [45]: Being another important CNN architecture, DenseNet offers the main advantage of alleviating the gradient vanishment problem with the direct connection of all the layers.

### 1.1 Related work in nuclear medical imaging for metastatic prostate cancer diagnosis in bones

Reviewing the relevant literature for diagnosis of bone metastasis using bone scintigraphy scans, the authors notice that only a couple of previous works have been adequately conducted for metastatic prostate cancer classification using CNNs, while the others are devoted to ANNs and their application in Computer-Aided Diagnosis (CAD). These works have investigated the use of Bone Scan Index (BSI), which was introduced to assess the bone scanning process and estimate the extent of bone metastasis [46, 47]. Specifically, it serves as a clinical, quantitative and reproducible parameter that can measure metastatic prostate cancer bone involvement [47]. The software developed for the BSI-based ANN approach, was EXINI bone (EXINI Diagnostics AB, Lund, Sweden) and afterwards, a revised version of this software, called BONENAVI (FUJIFILM Toyama Chemical, Co. Ltd, Tokyo, Japan), was engineered using a large number of Japanese multicenter training databases [48].

The first of the reported studies was devoted to the development of a classification algorithm based on CNNs for bone scintigraphy image analysis [49]. It was carried out as a master thesis in Lund University and is focused mainly on classification problems, without considering any identification and segmentation tasks. The used dataset was provided by Exini Diagnostics AB, in the form of image patches of already found hotspots. The process in which hotspots were segmented, cropped and collected from bone scans, was implemented using a software developed at Exini. BSI was calculated for whole-body bone scans, by segmenting the entire skeleton from the background in both the anterior and posterior views. Due to time frame restrictions, only the hotspots found in the spine have been used to train the CNN, since they were considered to be the easiest to classify. A shape model based on a mean shape of several normal whole-body scans, was fitted to the skeleton, using an image analysis algorithm, called Morphon registration. The outcomes of the aforementioned thesis [49] have shown that the calculated accuracy of the validation set was 0.875, whereas the calculated accuracy of the testing set was 0.89.

The second study explored CNNs for classification of prostate cancer metastases using bone scan images [50]. The tasks of this master project appeared to have a significant potential on classifying bone scan images obtained by Exini Diagnostics AB too, including BSI. The two tasks were defined as: i) classifying anterior / posterior pose and ii) classifying metastatic / non-metastatic hotspots. The outcome of this study is that the trained models produce highly accurate results in both tasks and they outperform other methods for all tested body regions in the case of metastatic / non-metastatic hotspots classification. The evaluation indicator of the area under Receiver Operating Characteristic (ROC) score was equal to 0.9739, which is significantly higher than the respective ROC of 0.9352, obtained by methods reported in the literature for the same test set.

The remaining research that concerns the same imaging modality (BS), is devoted to the introduction of CAD systems with the use of ANN and other Machine Learning (ML) methods for bone metastasis detection in bone scintigraphy images. Sadik et al. were the first to develop an automated CAD system as a clinical quality assurance tool, for the interpretation of bone scans [51–53]. This bone scan CAD software was trained to interpret bone scans using training databases that consist of bone scans from European patients who have the desired image interpretation, metastatic disease or not. The results showed a sensitivity of 90% at a specificity of 74%. These works result in certain outcomes that refer to the development of a totally automated computer-assisted diagnosis system that can identify metastases after examining bone scans, applying multi-layer perceptron ANN techniques, involving a small database of whole-body bone scans (135 patients). The highest sensitivity that was achieved from all the studies and accomplished during this thesis, was approximately 89% [54].

Horikoshi et al. compared the diagnostic accuracy of two CAD systems, one based on a European and another on a Japanese training database, in a group of bone scans from Japanese patients [48]. The Japanese CAD software showed a higher specificity and accuracy compared to the European. Comparing the sensitivities, the Japanese CAD software achieved 90%, whereas the European CAD software reached 83% [48].

In another study conducted by Tokuda et al., the diagnostic capability of a completely automated CAD system, which detects metastases in the images of bone scans by focusing on two different patterns, was investigated [55]. The first pattern was devoted to the detection of metastases per region; the second one detects metastases per patient. The investigated system was called “BONENAVI version 1”. The produced results have shown that the new CAD system is able to decrease the number of false positive findings, which depends on the primary lesion of cancer.

In 2016, Aslantas et al. proposed “CADBOSS” as a fully automated diagnosis system for bone metastases detections, using whole-body images [56]. The proposed CAD system combines an active contour segmentation algorithm for hotspots detection, an advanced method of image gridding to extract certain characteristics of metastatic regions, as well as an ANN classifier for identifying possible metastases. The calculated accuracy, sensitivity, and specificity of CADBOSS were 92.30%, 94%, and 86.67%, respectively, outperforming other state of the art CAD systems.

Additionally, ML methods have been exploited and applied in CAD systems for bone metastasis detection in bone scintigraphy images. A parallelepiped classification method was specifically deployed in [57] to assist physicians in bone metastases detection of cancer. Decision Trees (DT) and Support Vector Machines (SVM) were exploited for predicting skeletal-related events in cancer patients with bone metastases, achieving higher accuracies with a smaller number of variables than the number of variables used in Linear Regression (LR). ML techniques can be also used to build accurate models to predict skeletal-related events in cancer patients with bone metastasis, providing an overall classification accuracy of 87.58% ± 2.25% [58].

As far as PET and PET/CT imaging techniques in nuclear medicine are concerned, there are some recent and prominent studies that apply the advantageous features of CNNs. In [59], deep learning has been applied for classification of benign and malignant bone lesions in [F-18]NaF PET/CT images. The authors in this work followed the VGG19 architecture for their network by employing 16 3×3 convolutional layers, followed by 2 fully connected layers and a softmax layer as final activation. The ImageNet database of natural images was further used to pre-train the network’s weights. In this way, the network first is trained on general image features and later is tuned using the lesion images and the physician’s scores. Taking a closer look at the results, it can be concluded that network’s performance was improved when it was trained to differentiate between definitely benign (score = 1) and definitely malignant (score = 5) lesions. The values of prediction metrics were 0.88, 0.90, 0.85 and 0.90, concerning the accuracy, sensitivity, specificity and positive predictive value, respectively.

Also, a CNN-based system was examined in a recent retrospective study, which included 3485 sequential patients who underwent whole-body FDG PET-CT [60]. The main purpose of the study was to detect malignant findings in FDG PET-CT examinations, while a neural network model, equivalent to ResNet24, was built. Additionally, Grad-CAM was employed to identify the part of the image on which the neural network used the largest information. The findings of the study showed that Grad-CAM reasonably highlighted the area of malignant uptake, allowing physicians to make a diagnosis. The same research team recently (2019) developed a CNN-based system that predicts the location of malignant uptake and further evaluated predictions accuracy [61]. A network model with configuration equivalent to ResNet24, was used to classify whole-body FDG PET images.

In the research work [62], a simple CNN-based system that predicts patient sex from FDG PET-CT images, was proposed. Specifically, 6462 consecutive patients have participated in the study and underwent whole-body FDG PET-CT. The CNN system was used for classifying these patients by sex. Another CNN-based diagnosis system for whole-body FDG PET-CT was developed in [63], that predicts whether physician’s further diagnosis is required or not. A thorough analysis of the results shows that the accuracy considering images of patients presenting malignant uptake and images of equivocal was 93.2±3.9% and 87.8±5.3%, respectively.

The task of segmentation with the use of deep learning models in skeletal scintigraphy images, has been discussed in more research studies. For example, in [64], the authors followed different approaches to convert convolutional neural networks, designed for classification tasks, into powerful pixel-wise predictors. Moreover, in [65], a deep-learning based segmentation method was developed using prostate-specific membrane antigen (PSMA) PET images and showed significant promise towards automated delineation and quantification of prostate cancer lesions. However, this research domain that involves bone scintigraphy segmentation with the inclusion of advanced deep neural networks has not been yet well established.

Although bone scintigraphy is extremely important for the diagnosis of metastatic cancer, it is clear that a small number of research works have been carried out, which presented only some preliminary results, while the advantageous and outstanding capabilities of CNNs have not been fully investigated. Reviewing the relevant literature, it appears that CNNs have not been sufficiently applied for the diagnosis of prostate cancer metastasis from whole body images.

### 1.2 Aim and contribution of this research work

CNN is an efficient deep learning network architecture that has recently found great applicability in the medical domain. It has shown excellent performance on medical image applications, including bone scintigraphy and nuclear medical imaging, and can offer a positive impact on diagnosis tasks.

Nowadays, the main challenge in bone scintigraphy, as being one of the most sensitive imaging methods in nuclear medicine, is to build an algorithm that automatically identifies whether a patient is suffering from bone metastasis or not, based on patient’s whole body scans. It is of utmost importance that the algorithm needs to be extremely accurate due to the fact that patients’ lives could be at stake. Deep learning algorithms whose potential lies in the fact that they can improve the accuracy of cancer screening, have been recently investigated for nuclear medical imaging analysis. Recent studies in BS and PET have shown that a deep learning-based system can perform as well as nuclear physicians do in standalone mode and improve physicians’ performance in support mode. Even though BS is extremely important for the diagnosis of metastatic cancer, there is currently no research paper regarding the diagnosis of prostate cancer metastasis from whole body scan images that applies robust and more accurate deep CNNs.

This research study investigates the application of a deep learning CNN to classify bone metastasis using whole body images of men who were initially diagnosed with prostate cancer. The proposed method employs different CNN-based architectures with data normalization, data augmentation and shuffling in the preprocessing phase. In the training phase, the backpropagation technique has been used for updating the weights as part of the optimization process. Finally, the network architecture is fine-tuned and the configuration that offers the best performance is selected to train the CNN model.

The scope of the current work entails the following two main components: First, this study introduces the CNN method into the diagnosis of bone metastasis disease, based on whole body images. In the second phase, the paper deals with the improvement of the existing CNN method in terms of both network architecture and hyperparameter optimization. The deployed process seemingly improves the diagnostic effect of the deep learning method, making it more efficient compared to other benchmark and well-known CNN methods, such as ResNet50, VGG16, GoogleNET, Xception and MobileNet [66, 67].

The innovations and contributions of this paper are well summarized as follows:

- The development and demonstration of a simple, fast, robust CNN-based classification tool for the identification of bone metastasis in prostate cancer patients from whole-body scans.
- The rigorous CNN hyper-parameter exploration for determining the most appropriate architecture for an enhanced classification performance.
- A comparative experimental analysis, utilizing popular image classification CNN architectures, like ResNet50, VGG16, GoogleNET, Xception and MobileNet.

This paper is structured in the following fashion: Section 2 presents the material and methods related to this research study. Section 3 presents the proposed solution based on CNN method for bone metastasis diagnosis for prostate cancer patients using whole body images. Section 4 shows the results of exploration analysis of CNNs in Red, Green and Blue (RGB) mode, for different parameters and configurations, thus providing the best CNN model for this case study. Furthermore, all the performed experiments with representative results are gathered in section 4, whereas section 5 provides a thorough discussion on analysis of results. Section 6 concludes the paper and outlines future steps.

## 2. Materials and methods

### 2.1 Patients and images

A retrospective review of 970 consecutive, whole body scintigraphy images from 817 different male patients who visited Nuclear Medicine Department of Diagnostic Medical Center “Diagnostico A.E.” in Larisa, Greece, from June 2013 to June 2017, was performed. The selection criterion was prostate cancer patients who had undergone whole-body scintigraphy, because of suspected bone metastatic disease.

Due to the fact that whole body scan images contain some artifacts and other non-related to bone uptake, such as urine contamination and medical accessories (i.e. urinary catheters) [68], as well as the frequent visible site of radiopharmaceutical injection [69], a preprocessing approach was accomplished to remove these artifacts and non-osseous uptake from the original images. This preprocessing method was accomplished by a nuclear medicine physician before the use of the dataset in the proposed classification approach.

**Fig 1:**
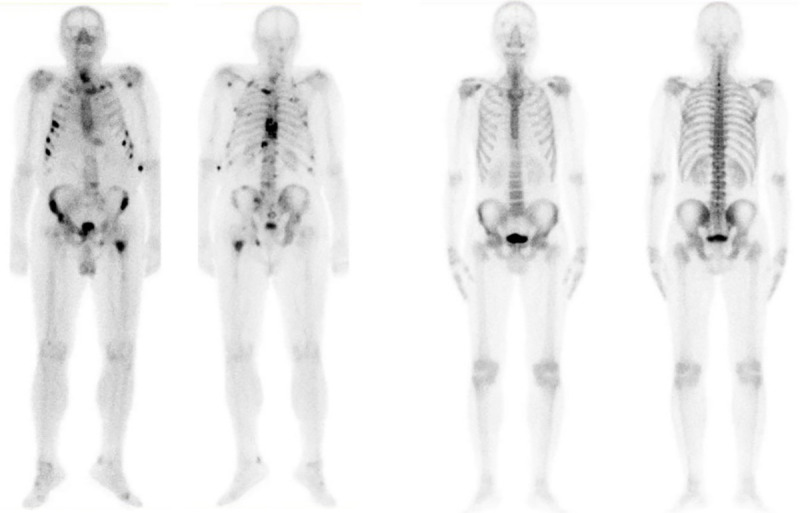
Image samples in the dataset (Label: (a) Metastasis is present or (b) absent).

The initial dataset of 970 images contained not only bone metastasis presence and absence patient’s cases suffering from prostate cancer, but also degenerative lesions [70]. Due to this fact, as well as aiming to cope with a two-class classification problem in this study, a pre-selection process, concerning images of healthy and malignant patients, was accomplished. In specific, 586 out of 970 consecutive whole-body scintigraphy images of men from 507 different patients were selected and diagnosed accordingly by a nuclear medicine specialist, with 15 years of experience in bone scan interpretation. Out of 586 bone scan images, 368 bone scans concern male patients with bone metastasis and 218 male patients without bone metastasis. A nuclear medicine physician classified all the patient cases into 2 categories: 1) metastasis absent and 2) metastasis present, which was used as a gold standard (see Fig 1). The metastatic images were confirmed by further examinations performed by CT/MRI.

### 2.2 Whole-body scintigraphy (Bone scans)

A Siemens gamma camera Symbia S series SPECT System (by dedicated workstation and software Syngo VE32B) with two heads and low energy high-resolution collimators was used for patients scanning. The speed of scanning was 12 cm/min with no pixel zooming. Two types of radionuclide were used for bone scintigraphy: 99m-Tc-HDP (TechneScan®) and 99-Tc-MDP (PoltechMDP 5mg). Whole body scintigraphy was acquired approximately 3 hours after intravenous injection of 600–740 MBq of radiopharmaceutical agent, depending on the patient body type. The common intravenous injection was 670 MBq of radiopharmaceutical agent.

In total, 586 planar bone scan images from patients with known prostate cancer were reviewed, retrospectively. The whole-body field was used to record anterior and posterior views digitally with resolution 1024 ×256 pixels. Images represent counts of detected gamma decays in each spatial unit with 16-bit grayscale depth.

The image data acquired were originally in DICOM files, a commonly used protocol for storage and communication in hospitals. These image data were extracted from these DICOM files to create new images in JPEG-format, instead. A novel dataset of bone scintigraphy images containing men patients suffering from prostate cancer with metastasis present and metastasis absent (two distinct classes of healthy and malignant cases), was prepared for experimentation. This dataset consisting of whole-body scans is available for research use after request.

This study was approved by the Board Committee Director of the Diagnostic Medical Center “Diagnostico-Iatriki A.E.” and the requirement to obtain informed consent was waived by the Director of the Diagnostic Center due to its retrospective nature. All procedures in this study were in accordance with the Declaration of Helsinki.

### 2.3 Methodology

**Fig 2:**
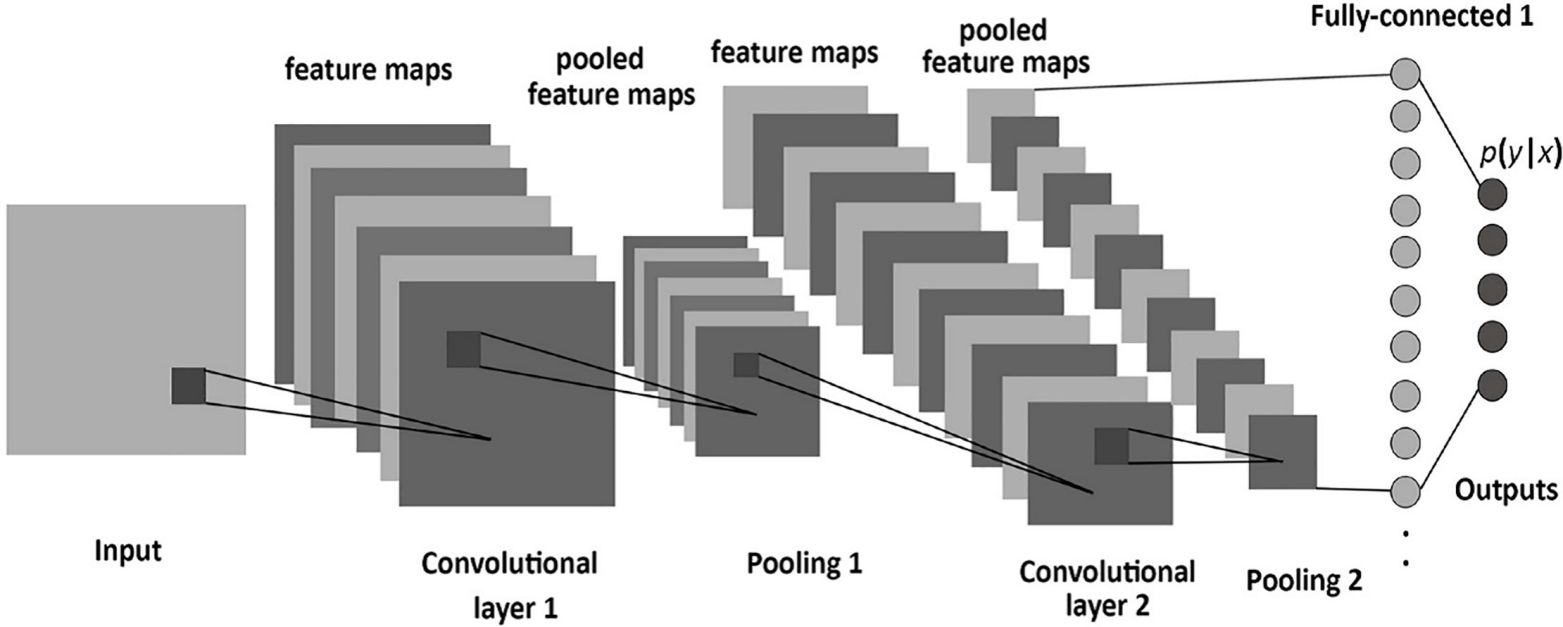
Flowchart of the proposed CNN methodology.

The problem of classifying bone metastasis images is a complex procedure and so, effective machine learning methods need to be exploited to cope with this diagnosis task. Deep learning methods such as CNNs are applied in order to train a classifier to distinguish images of prostate cancer patients with bone metastasis, and metastasis absent on healthy patients. The effective CNN method for bone metastasis classification proposed in this paper, includes three processing steps: data pre-processing for the collected scan data normalization, a training phase for CNN learning and validation, and testing which includes the evaluation of the classification results, as illustrated in Fig 2. The proposed methodology is thoroughly presented in the following sections.

#### 2.3.1 Data pre-processing

*Step 1*: *Load images to RGB*. The original images were saved in RGB mode. All images are stored in a respective folder before loaded into the computer memory for CNN training. In each image, a suitable prefix was defined according to the patient’s category, for example “malignant_” and “healthy_”. Next, a small script was set up in order to assign a numerical value as label to each image, according to its prefix. In our case, the value ‘0’ was assigned for “malignant_” prefixes, whereas the value ‘1’ for “healthy_” prefixes.

*Step 2*: *Data normalization*. It is common to follow a normalization process (feature scaling) in most machine learning algorithms [70]. The min-max normalization processnormalizes the dataset values within the (0,1) to ensure that all feature data are in the same scale for training and testing. The data normalization process also assists the convergence of the backpropagation algorithm.

*Step 3*: *Data shuffle*. To avoid or eliminate unbiased sampling in machine learning, an appropriate shuffling method is needed to be defined. More specifically, a random-number generator is used to reorder the images. An image sample, chosen randomly, is meant to be an impartial representation of the total images. An unbiased random sample is important for machine learning to provide reliable conclusions. In this study, Python’s random.shuffle method is used for dataset shuffling.

*Step 4*: *Data augmentation*. Data augmentation is used as a method to artificially increase the diversity of training data by a large margin, by manipulating the existing data instead of creating new. Data augmentation techniques such as cropping, padding, and horizontal flipping are commonly used to train large neural networks [71]. In this research, the number of images used for learning processes was followed by an image augmentation processing such as rotation, enlargement/reduction, range and flip. Note that the original images used for the test were not subjected to such an augmentation process.

*Step 5*: *Data split*. The dataset was split into three sections, a training portion, a validation portion that would allow the training process to improve, and a testing (hold-out) portion, which is part of the dataset that is completely hidden from the training process. The first data split takes place by removing 15% of the total dataset and saving for later use as testing. The remaining 85% of the dataset is then split again into an 80/20 ratio, where the small portion is used as validation set. The validation set is used during the training process in order to help the algorithm update its weights appropriately. Thus, it improves its performance and avoids overfitting. The testing dataset is used to verify if the unknown to the model data have been classified correctly

#### 2.3.2 Convolutional neural networks–CNNs (Training and validation)

**Fig 3:**
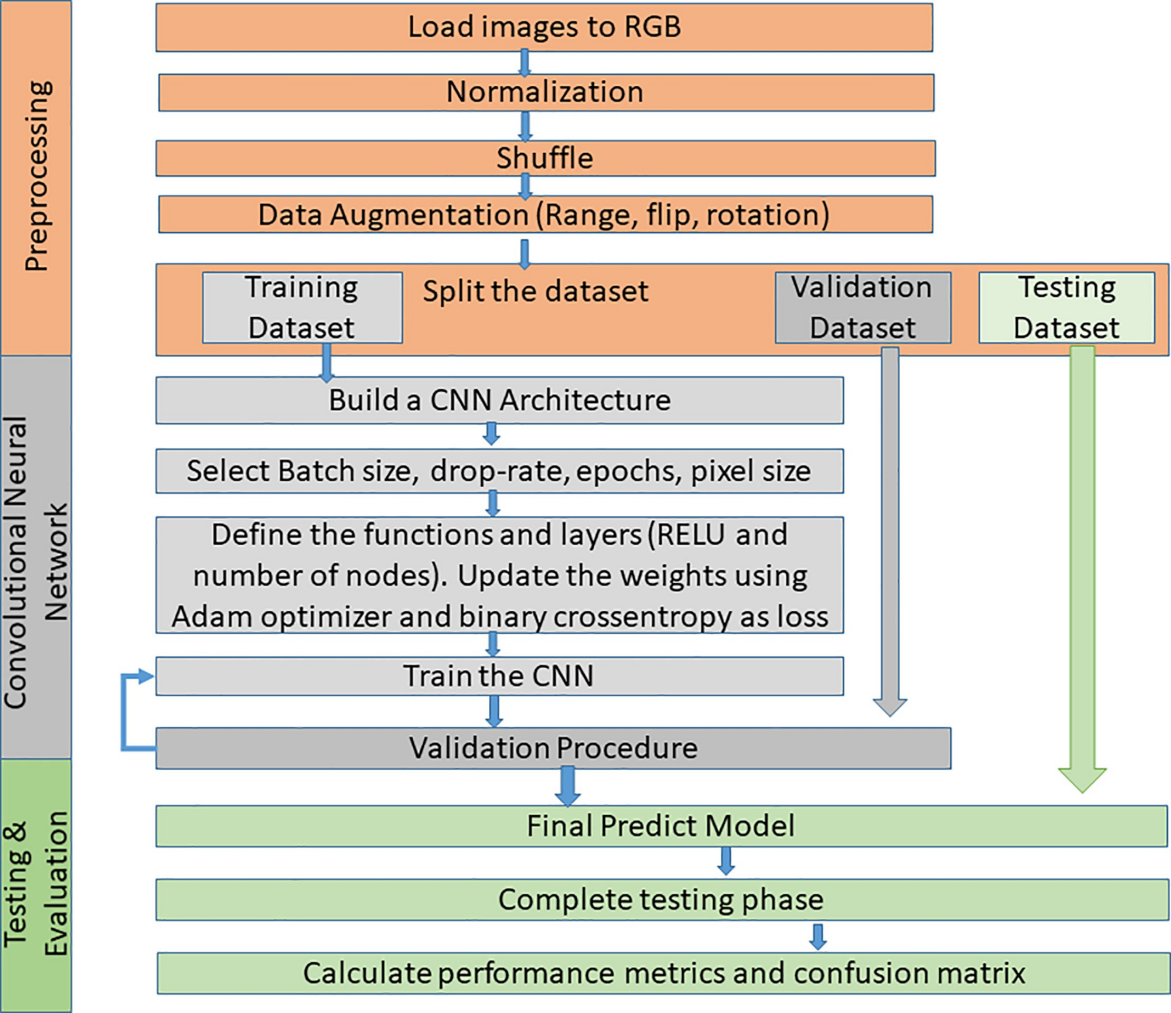
Architecture of a convolutional neural network [72].

To define a proper architecture of a Convolutional Neural Network (CNN) for image classification, an exploration process is needed. In the experimentation and trial phase, the most important parameters that can lead to effective network architecture were explored; the number of convolutional layers, the number of pooling layers, the number of nodes in the dense layers, the dropout rate and the batch size. In S1 Appendix, the main aspects of CNN implementation are provided. A typical CNN architecture is illustrated in Fig 3.

Next, a number of functions that characterize the CNN architecture is being defined through bibliographic research. The main functions include the activation function, which is the function that defines the output of the layer and the loss function, which is the function that is used for the optimization of the network’s weights. Further explanation is provided in section 2.4.

The training process takes place when the algorithm tries to create a function that describes the desired relation, based on the training data. It then makes predictions based on this function and moves to the validation step. The validation dataset is used for the validation process in which, the main aim isthe error minimization, that reveals the prediction efficiency of the proposed method.The minimization of the training loss and the validation loss are also desired outcomes for the validation phase to further demonstrate the model performance.

#### 2.3.3 Testing and evaluation

The testing process for the CNN model is accomplished by using the testing data, which are completely unknown to the model. The classifier makes predictions on each image class and finally compares the calculated predicted class with the true class. After a classifier is trained, it can predict the class of a new entry from the testing set. Depending on the prediction and the actual class it belongs, this prediction can be true positive (TP) or true negative (TN), if it is classified correctly, or false positive (FP) or false negative (FN) if it is misclassified. Next, for classifier evaluation, some well-known and popular performance metrics, such as testing accuracy, precision, recall, sensitivity, specificity and F1-score of the model are computed [16, 19, 73] (see S2 Appendix). An error/confusion matrix is also employed to further evaluate the performance of the model. It consists of rows and columns, which show the correctly and wrongly classified images, in each column. Calculating the confusion matrix can give us a better view of the proposed classification model.

## 3. Proposed CNN architecture

In this research study, a CNN architecture is proposed to precisely identify bone metastasis from whole-body scans of men suffering from prostate cancer. The developed CNN will prove its capability to provide high accuracy with a simple and fast architecture for whole-body image classification. Through an extensive CNN exploration process, we conducted experiments with different values for our parameters, like pixels, epochs, drop rate, batch size, number of nodes and layers [25]. In common classic feature extraction techniques, a manual feature selection was required to extract and utilize the appropriate feature. Convolutional Neural Networks (CNN), resembling in type Artificial Neural Networks (ANN), can perform feature extraction techniques automatically by applying multiple filters on the input images and then, through an advanced learning process, they select those that have the highest impact on the images classification.

In this research study, a deep-layer network with 3 convolutional—pooling layers, 1 dense layer followed by a dropout layer, as well as a final output layer with one node, is built. The dedicated problem is binary classification. The suggested network architecture along with the layers shapes and the number of trainable parameters are given in Table 1.

**Table 1:** The input vector concepts of each scenario.

	Layers	Output Size	Description
	Batch Size: 16, Epochs: 200, pixel size 256x256x3, dropout: 0.7		
Layer1	Convolution	(None,256,254,8)	Filters: 8, Kernel Size: 3x3,Input size: 256x256x3, Activation: ReLU
	Pooling	(None, 127, 127,8)	2x2 Max Pooling
	Dropout	(None, 127, 127,8)	Drop Rate = 0.7
Layer2	Convolution	(None, 125, 125,8)	Filters: 16, Kernel Size: 3x3, Activation: ReLU
	Pooling	(None,62,62,16)	2x2 Max Pooling
	Dropout	(None,62,62,16)	Drop Rate = 0.7
Layer3	Convolution	(None,60,60,32)	Filters: 32, Kernel Size: 3x3, Activation: ReLU
	Pooling	(None,30,30,32)	2x2 Max Pooling
	Dropout	(None,30,30,32)	Drop Rate = 0.7
	Flatten	(None,2312)	
	Dense	(None,28800)	64 nodes, Activation: ReLU
	Dropout	(None,64)	Drop Rate = 0.7
	Dense	(None,1)	1 node, Activation: Sigmoid

The images enter the network at various pixels dimensions, starting from 100x100 pixels to 300x300 pixels. Following the structure of the CNN, the first (input) convolutional layer consists of 3 filters (kernels) of size 3x3, always followed by a max-pooling layer of size 2x2 and a dropout layer with 0.7 as dropout rate. Each next convolutional layer doubles the numbers of filters (8), just like the following max-pooling layers. Next, a flattening operation transforms the 2-dimensional matrices to 1-dimensional arrays, in order to run through the hidden fully connected (dense) layer with 64 nodes. To avoid overfitting, a dropout layer was suggested to drop randomly 70% of the learned weights. The final layer is a signle-node layer (output layer).

In most CNN models, the rectified linear unit (ReLU) function is the activation function that is used in all convolutional and fully connected (dense) layers, whereas the sigmoid function is the final activation function used in output nodes. The trials for algorithm run were accomplished for different number of epochs (100, 200, 300, 500), aiming at exploiting the most appropriate number of epochs for CNN training. Moreover, two performance metrics, concerning accuracy and loss, are used. As it regards loss, the binary cross-entropy function is calculated with an ADAM optimizer. For model training, the ImageDataGenerator class from Keras was used, offering augmentations on images like rotations, shifting, zoom, flips and more.

## 4. Results

This section reports on the results produced in this research study, which is devoted to the use of whole-body scans from SPECT, to perform the diagnosis of bone metastasis for patients suffering from prostate cancer. Each process was repeated 10 times to calculate the classification accuracy.

### Hardware and software environments

This experiment was performed in a collaboratory environment, called Google Colab [74], which is a free Jupyter notebook environment in the cloud. The main reason for selecting this cloud environment of Google Colab is that supports free GPU acceleration. The frameworks Keras 2.0.2 and TensorFlow 2.0.0. were used, as well as python language 3.7, CNN with structure (Convolution layer, 3; Maxpooling layer, 3) and Adam Optimizer. Other CNN methods, including VGG16 [33], ResNet50 [38], Inception V3 [67], Xception [75], and MobileNet [76] were also implemented, so as a comparative analysis could be performed between them and the method proposed in this paper.

OpenCV was used for loading and manipulating images, Glob for reading filenames from a folder, Matplotlib for plot visualizations and finally, Numpy for all mathematical and array operations. Python was used for coding, with the CNN being programmed with Keras (with Tensorflow [77]), whereas, data normalization, data splitting, confusion matrices and classification reports were carried out with Sci-Kit Learn. The computations ranged between 2’ to 4’ per training (epoch) for RGB images (256x256x3), depending on the different input.

It is noteworthy that the original images, as acquired from the scanning device, are in RGB format, containing 3-channel color information. Even though the images are in RGB structure, they appear as grayscale due to the absence of color components in the image. Regarding the type of CNN, we applied a 2D CNN for each color channel of each image, and then aggregated the inputs into the final part of the network, which is comprised by a dense layer.

Following the steps described in section 2.3, the images are initially loaded in RGB mode, by default. Normalization, shuffling and data augmentation are followed just before the training phase. Next, the dataset is split into training, validation and testing and afterwards, images are passed through the proposed CNN network that has been pre-trained on the ImageNet data set [78]. This data set is able to provide another, more efficient method for weights initialization, which seems of high utility, in any image-related task.

The classification task was two-fold, one for bone metastasis presence and another one for metastasis absence in patients suffering from prostate cancer, considering in total, 586 samples of men patients. For the purposes of this study, we employed different CNN-based architectures and hyper-parameter selection, as defined in Section 2.

**Fig 4:**
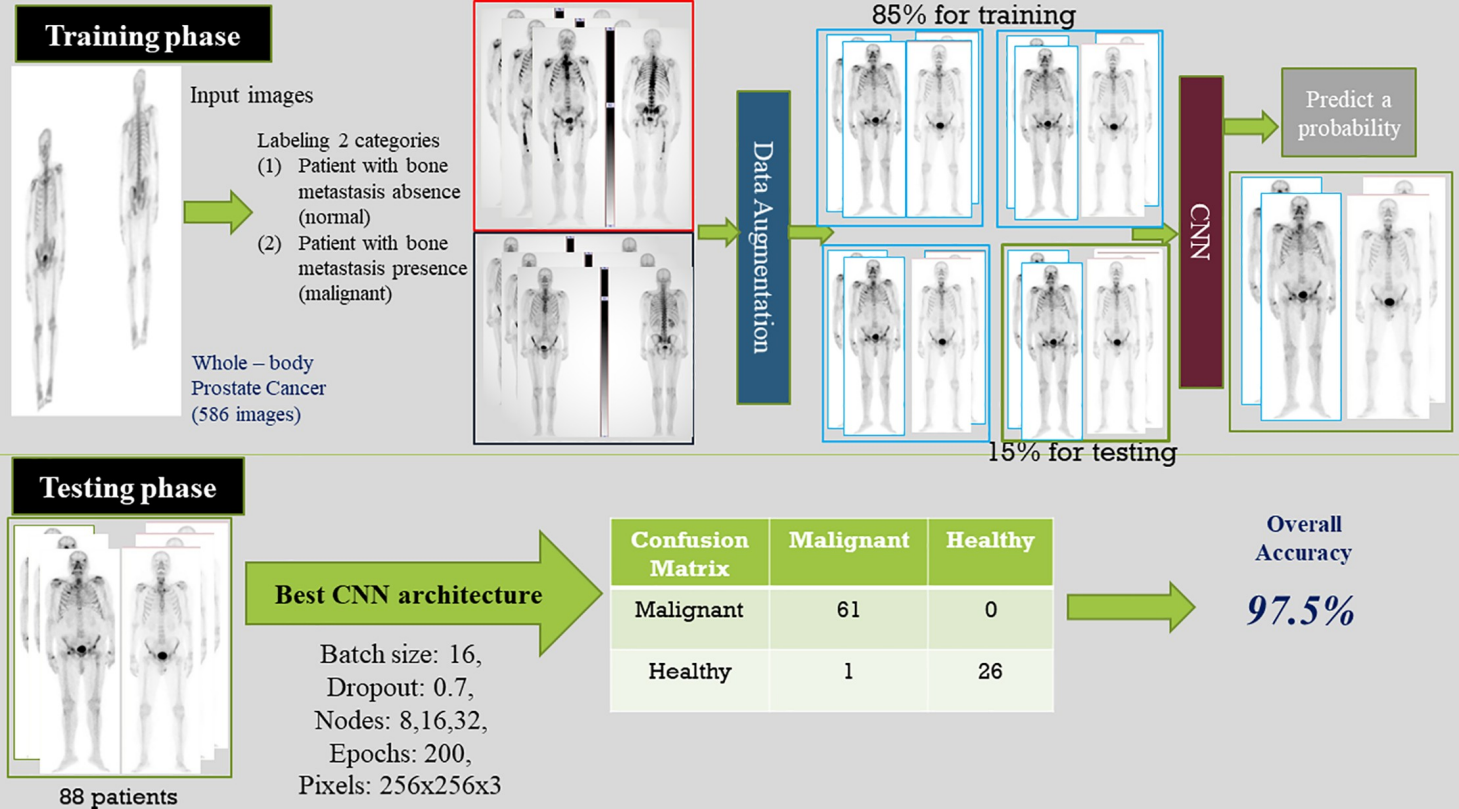
Overview of training and testing phase of CNN based whole-body image analysis.

The whole classification process consisting of these two phases, is illustrated in the following Fig 4, depicting an overall view of the training and testing phase for the examined dataset of 586 patients by classifying them in two categories.

A meticulous CNN exploration process was accomplished, in which we conducted experiments with various convolutional layers, drop rates, epochs, number of dense nodes, pixel sizes and batch sizes. Different values for image pixel sizes were examined, such as 100×100x3, 200×200x3, 256×256x3, 300×300x3 and 350x350x3, as well as various values for batch sizes, such as 8, 16, 32 and 64 were investigated. In addition, variant drop rate values were studied, for example 0.2, 0.5, 0.7 and 0.9 and a divergent number of dense nodes, like 16, 32, 64 and 128 were explored. The number of epochs that was explored ranged from 100, 200, 300 to 500. The number of convolutional and pooling layers was also investigated.

Notable is the fact that we performed many experiments with several convolutional layers, epochs and pixel sizes, to find the optimum values. We observed that for epochs = 200 and for pixel sizes (256,256,3), the CNN models with three convolutional layers had the best performance concerning the classification accuracy and sensitivity. After a thorough CNN exploration analysis, we concluded that the best CNN configuration was the following: a CNN with 3 convolutional layers, starting with 8 filters for the first layer, and for each convolutional layer that comes next, the number of filters is doubled (8->16->32). The same logic is followed for the following max-pooling layers. All filters have dimensions of 3×3. This set-up is shown in Table 1. After carefully selecting the CNN architecture along with the number of epochs and pixel sizes (epochs = 200 and pixel = 256×256x3), we conducted further experiments with varying drop rates and batch sizes, to find out the best performance parameters.

Table 2 gathers the performance analysis and results for the CNN models with 3 convolutional layers (8,16,32), epochs = 200, dropout = 0.7, pixel size = 256x256x3, dense nodes = 64 and different batch sizes (in Google Colab [74]) for 10 runs, whereas Table 3 presents the 5 runs of the best CNN architecture, entailing 3 convolutional layers (8,16,32), epochs = 200, dropout = 0.7, pixel size = 256x256x3, batch size = 16 and different dense nodes.

**Table 2:** CNN model with 3 conv (8,16,32), epochs = 200, dropout = 0.7, pixel size = 256x256x3, dense nodes = 64 and different batch sizes.

	batch size = 8				batch size = 16				batch size = 32			
	Acc. Val	Loss Val	Acc Test	Loss Test	Acc. Val	Loss Val	Acc Test	Loss Test	Acc. Val	Loss Val	Acc Test	Loss Test
**Run 1**	96,88	0,09	95,45	0,12	100,00	0,04	97,50	0,09	95,83	0,12	95,31	0,125
**Run 2**	95,83	0,09	97,72	0,14	96,88	0,09	96,25	0,08	97,91	0,076	96,875	0,107
**Run 3**	97,91	0,10	96,59	0,07	94,79	0,15	97,50	0,08	97,91	0,103	96,875	0,103
**Run 4**	97,92	0,06	96,59	0,08	95,83	0,17	98,75	0,11	94,79	0,126	98,43	0,074
**Run 5**	96,88	0,11	96,59	0,09	94,79	0,16	98,75	0,06	98,95	0,08	95,31	0,135
**Run 6**	95,83	0,10	98,86	0,08	97,92	0,10	96,25	0,15	95,83	0,218	90,625	0,293
**Run 7**	93,75	0,14	96,59	0,17	95,83	0,12	97,50	0,07	97,91	0,073	96,875	0,114
**Run 8**	94,79	0,11	95,45	0,11	95,83	0,13	97,50	0,05	95,83	0,2	98,43	0,059
**Run 9**	97,92	0,08	97,72	0,08	98,43	0,07	96,25	0,14	96,87	0,088	96,875	0,107
**Run 10**	97,92	0,77	95,45	0,09	97,20	0,07	97,50	0,06	98,95	0,077	93,75	0,114
**Average**	96,56	0,16	96,70	0,10	96,75	0,11	97,38	0,09	97,08	0,12	95,94	0,12

**Table 3:** CNN model with 3 conv (8,16,32), epochs = 200, dropout = 0.7, pixel = 256x256x3, different dense nodes and batch size = 16.

	Dense nodes = 32				Dense nodes = 64				Dense nodes = 128			
	Acc. Val	Loss Val	Acc Test	Loss Test	Acc. Val	Loss Val	Acc Test	Loss Test	Acc. Val	Loss Val	Acc Test	Loss Test
**Run 1**	97,92	0,08	95,00	0,16	100,00	0,04	97,50	0,09	97,92	0,1	98,75	0,05
**Run 2**	96,87	0,12	97,50	0,09	96,88	0,09	96,25	0,08	98,96	0,05	97,5	0,09
**Run 3**	98,96	0,06	97,50	0,11	94,79	0,15	97,50	0,08	96,87	0,09	96,25	0,09
**Run 4**	96,87	0,07	98,75	0,09	95,83	0,17	98,75	0,11	97,92	0,05	98,75	0,06
**Run 5**	97,92	0,06	96,25	0,13	94,79	0,16	98,75	0,06	95,83	0,12	93,75	0,14
**Average**	97,71	0,08	97,00	0,12	96,46	0,12	97,75	0,08	97,5	0,08	97	0,09

Table 4 gathers the values of the performance metrics for the set of the most accurate and robust CNN models, which consists of 3 conv (8,16,32), epochs = 200, dropout = 0.7, pixel size = 256x256x3, various dense nodes (32,64,128) for best batch size 16 and various batch sizes for the best number of dense nodes which is 64. Overall, it is observed that the CNN model with batch size 16 and 64 dense nodes has the highest classification accuracy and sensitivity, as well as the minimum loss. S1 Table shows analytically the calculated values of the performance metrics for the best CNN network for 10 runs (batch size = 16, dropout = 0.7).

**Table 4:** Evaluation metrics for the set of CNN models consists of 3 conv (8,16,32), epochs = 200, dropout = 0.7, pixel size = 256x256x3, various dense nodes and batch sizes.

		Batch size = 16			Dense nodes = 64	
		Dense nodes			Batch size	
		32	64	128	8	32
**Accuracy**		97	97.75	97	96.70	95.94
**Loss**		0,12	0.08	0,09	0.10	0.12
**Precision**	**Malignant**	0.966	0.987	0.872	0.98	0.96
	**Healthy**	0.976	0.947	0.85	0.947	0.972
**Recall**	**Malignant**	0.984	0.973	0.867	0.966	0.974
	**Healthy**	0.946	0.975	0.854	0.965	0.94
**F1-Score**	**Malignant**	0.976	0.98	0.87	0.972	0.966
	**Healthy**	0.958	0.96	0.848	0.955	0.954
**Sensitivity**	**Malignant**	0.968	0.984	0.872	0.98	0.959
	**Healthy**	0.974	0.945	0.847	0.946	0.966
**Specificity**	**Malignant**	0.974	0.95	0.849	0.946	0.97
	**Healthy**	0.968	0.987	0.872	0.981	0.932
**Execution Time (s)**		905	958	979	1008	927

**Fig 5:**
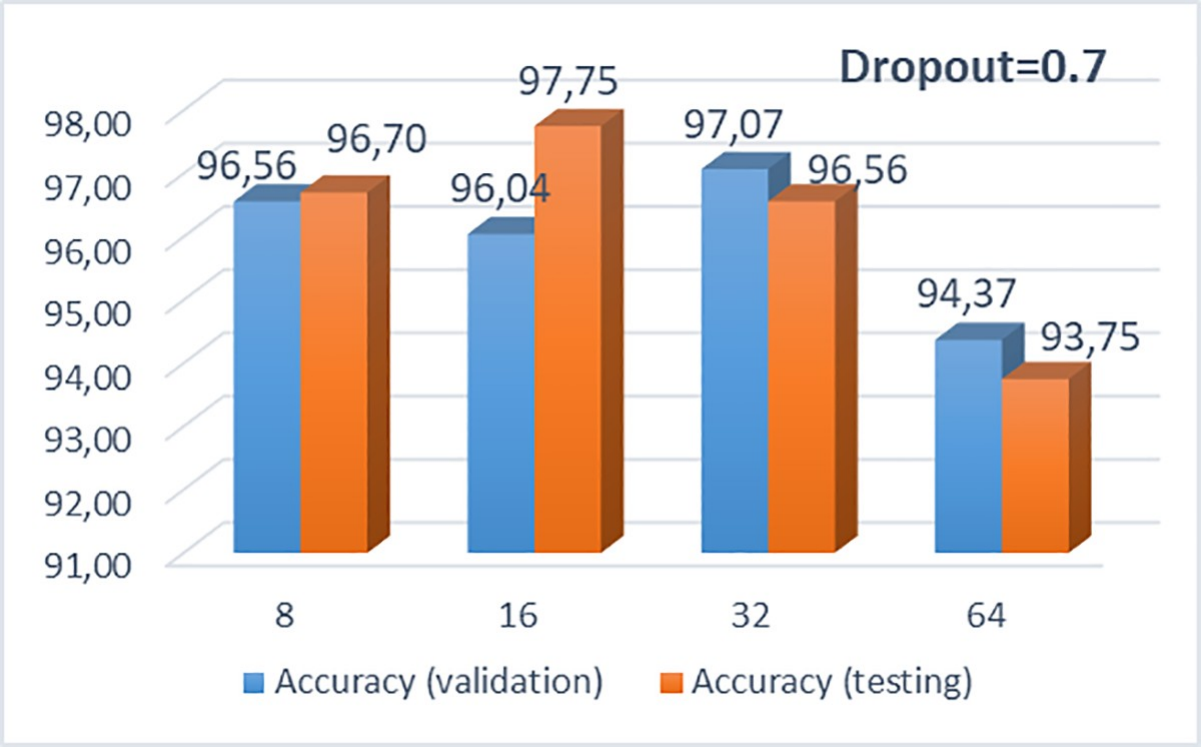
Bar chart with calculated accuracies for various batch sizes (8, 16, 32, 64) and dropout = 0.7.

**Fig 6:**
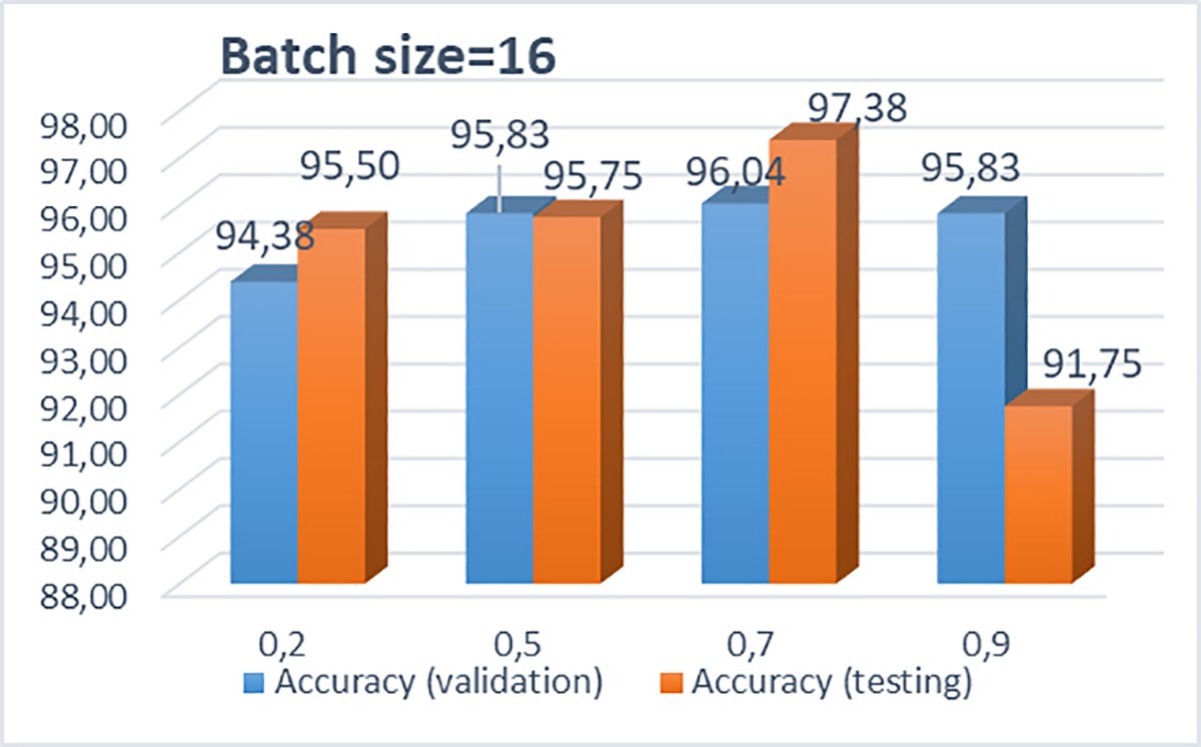
Bar chart with calculated accuracies for various dropouts (0.2, 0.5, 0.7 and 0.9) and batch size = 16.

In what follows, Figs 5 and 6 illustrate the performance of the proposed CNN architecture of 3 convolutional layers, 256x256x3 pixels, 200 epochs and 64 dense nodes, for different drop rates and different batch sizes, respectively. It is clearly observed that the CNN model with the optimum performance, concerning the testing accuracy, is the model with dropout = 0.7 and batch size = 16. In S2, S3 and S4 Tables, we provide some results produced after further simulations for different drop rates and batch sizes for the selected pixel size. Also, some indicative results with different pixel sizes (300, 300, 3) and another efficient CNN architecture, consisting of four convolutional layers (8,16,32,64), epochs = 200, dropout = 0.7, pixel = 256x256x3, various dense nodes and the best batch size 16 are depicted in S5 and S6 Tables, respectively.

For the best dropout rate (0.7) and the best batch size (16), the accuracies for various dense nodes (32, 64 and 128) are depicted in Table 3. It is observed that these CNN models, with dense nodes 32, 64 and 128, provide similar classification accuracies, with minimum testing loss. The most accurate CNN model with respect to classification accuracy, sensitivity and loss, is the CNN with dropout 0.7, batch size 16 and 64 dense nodes. The best confusion matrix for the proposed CNN model, considering malignant (metastasis) and benign (no metastasis) patients, is shown in Table 5.

**Table 5:** Best confusion matrix for the proposed CNN.

	*Malignant*	*Benign*
***Malignant***	61	0
***Benign***	1	26

**Fig 7:**
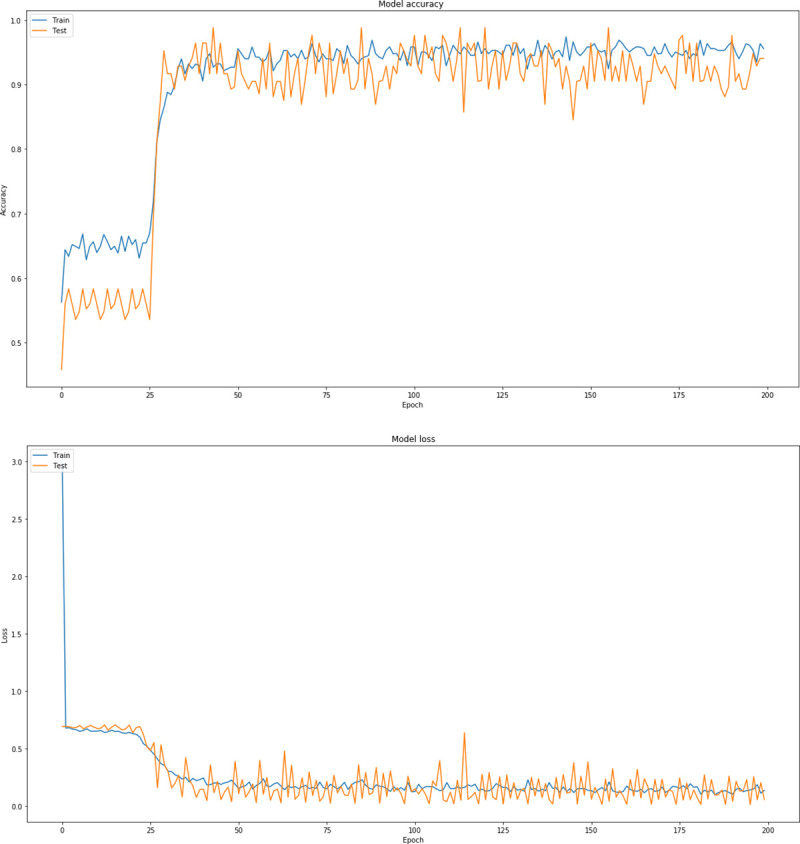
Precision curves for best CNN architecture with dense = 64, dropout = 0.7, in RGB mode: (a) Accuracy and (b) loss.

**Fig 8:**
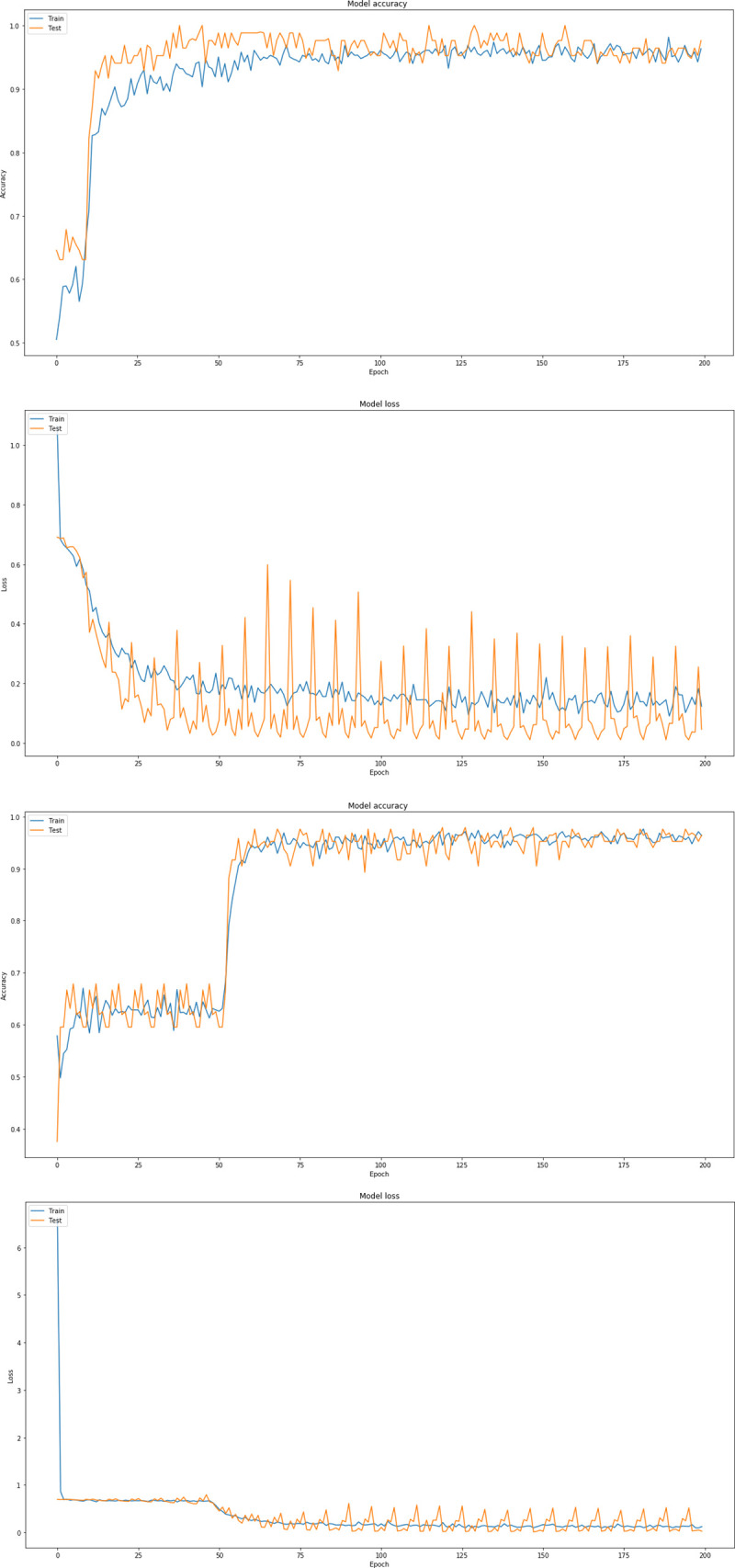
Precision curves for best CNN architectures with dense = 32, dropout = 0.7, in RGB mode: (a) Accuracy and (b) loss, and dense nodes = 128, (c) Accuracy and (d) loss.

Through this extensive CNN exploration, it is summarized that the best CNN model has the following characteristics: batch-size = 16, dropout = 0.7, nodes (3 conv layers) = 8, 16, 32, Dense Nodes = 64, Epochs = 200 and Pixel = (256, 256, 3). Fig 7 represents the precision curves of testing accuracy and loss for the CNN model that performed the best, whereas Fig 8 illustrates the precision curves for the other two CNN architectures, with best dropout = 0.7 and best batch size 16, considering 32 and 128 dense nodes, respectively.

To further investigate the performance of the proposed CNN architecture, an extensive comparative analysis between the state of the art CNNs, such as VGG16 [43, 44], ResNet50 [38], Inception V3 [67], Mobile Net [76] and Xception [75] and our best model, was performed. The following well-known CNN architectures were used: (i) ResNet50, which is a 50 weight layer deep version of ResNet (Residual neural Network), with 152 layers based on “network-in-network” micro-architectures [38]. ResNet has less parameters than the VGG network, demonstrating that extremely deep networks can be trained using standard SGD (and a reasonable initialization function) through the use of residual modules. (ii) VGG16 [43, 44], which is an extended version of VGG (Visual Geometry Group) as it contains 16 weight layers within the architecture. VGGs are usually constructed by using 3×3 convolutional layers, which are stacked on top of each other.

Inception V3 [67], which is Inception’s third installment, includes new factorization ideas. It is a 48-layers deep network which incorporates RMSProp optimizer and computes 1×1, 3×3, and 5×5 convolutions within the same module of the network. Its original architecture is GoogleNet. Szegedy et al. proposed an updated version of the architecture of Inception V3, which was included in Keras’ inception module [67]. This updated version is capable to further boost the classification accuracy of ImageNet. Xception, which is an extension of the Inception architecture, replaces the standard Inception modules with depth-wise separable convolutions [75]. MobileNet convolutes each channel separately instead of combining and flattening them all, with the use of depth-wise separable convolutions [76]. Its architecture combines convolutional layers, depth-wise and point-wise layers to a total number of 30. These popular CNNs were used for transfer learning with the weights from the ImageNet [78] dataset.

In this research work, after an extensive exploration with the provided architectures of popular CNNs, the following parameters were defined, regarding the well-known CNNs. Table 6 depicts the optimum parameters for the five well-known CNNs (VGG16, ResNet50, MobileNet, Inception V3 and Xception), compared with the proposed best performed CNN. Average running time was calculated for each CNN architecture for all models and is also presented in Table 6. It is obvious that the proposed CNN model is less time-consuming, providing at the same time higher classification accuracy, when compared with all the other popular CNN models.

**Table 6:** Selected values of parameters for benchmark CNNs.

CNNs	Epochs, pixel size, dropout, batch size, dense nodes,	Average Running Time for 200 epochs
**Best CNN**	Batch-size = 16, dropout = 0.7, nodes [3 conv layers) = 8,16,32, Dense Nodes: 64, Epochs:200, Pixel = [256x256x3)	418 sec
**VGG16**	Pixel size (256x256x3), batch size = 64, dropout = 0.2, dense nodes 2x512, epochs = 200	1005 sec
**ResNet50**	Pixel size (300x300x3), batch size = 8, dropout = 0.2, Global Average Pooling, dense nodes 512x512, epochs = 200	2434 sec
**MobileNet**	Pixel size (300x300x3), batch size = 16, dropout = 0.2, global average pooling, epochs = 200	1380 sec
**Inception V3**	Pixel size (250x250x3), batch size = 16, dropout = 0.7, dense nodes = 1500x1500, epochs = 200	1317 sec
**Xception**	Pixel size (300x300x3), batch size = 8, dropout = 0.7, Global Average Pooling, dense nodes = 512x512, epochs = 200	3532 sec

In what follows, Tables 7, 8 and 9 gather the results of the state-of-the-art CNN models, which are straightforward compared with our best performed CNN configuration, suggested in this research work.

**Table 7:** Accuracy and Loss (validation and testing) of the proposed CNN model and benchmark CNNs.

Average	Proposed CNN	ResNet50	VGG16	MobileNet	Inception V3	Xception
**Accuracy (Validation)**	96.04	95	93.44	97.29	95.52	97.5
**Loss (Validation)**	0.13	0.12	0.17	0.07	0.12	0.08
**Accuracy (Testing)**	97.38	94.98	93.75	98.13	96.7	96.36
**Loss (Testing)**	0.087	0.16	0.17	0.06	0.14	0.13

**Table 8:** Malignant disease class performance comparison of different methods.

Network	Proposed CNN	ResNet50	VGG16	MobileNet	Inception V3	Xception
**Accuracy**	97,38	94.98	93.75	98.13	96.7	96.36
**Loss**	0.087	0.16	0.17	0.06	0.14	0.13
**Precision**	0.987	0.938	0.922	0.986	0.951	0.942
**Recall**	0.973	0.962	0.935	0.981	0.97	0.99
**F1-score**	0.98	0.948	0.922	0.983	0.958	0.968
**Sensitivity**	0.984	0.939	0.921	0.986	0.952	0.941
**Specificity**	0.95	0.937	0.869	0.965	0.955	0.99

**Table 9:** Healthy class performance comparison of different methods.

Network	Proposed CNN	ResNet50	VGG16	MobileNet	Inception V3	Xception
**Accuracy**	97.38	94.98	93.75	98.13	96.7	96.36
**Precision**	0.947	0.938	0.87	0.966	0.954	1
**Recall**	0.975	0.9	0.832	0.973	0.908	0.872
**F1-score**	0.96	0.912	0.835	0.968	0.927	0.926
**Sensitivity**	0.945	0.937	0.921	0.973	0.953	1
**Specificity**	0.987	0.939	0.921	0.983	0.953	0.941

**Fig 9:**
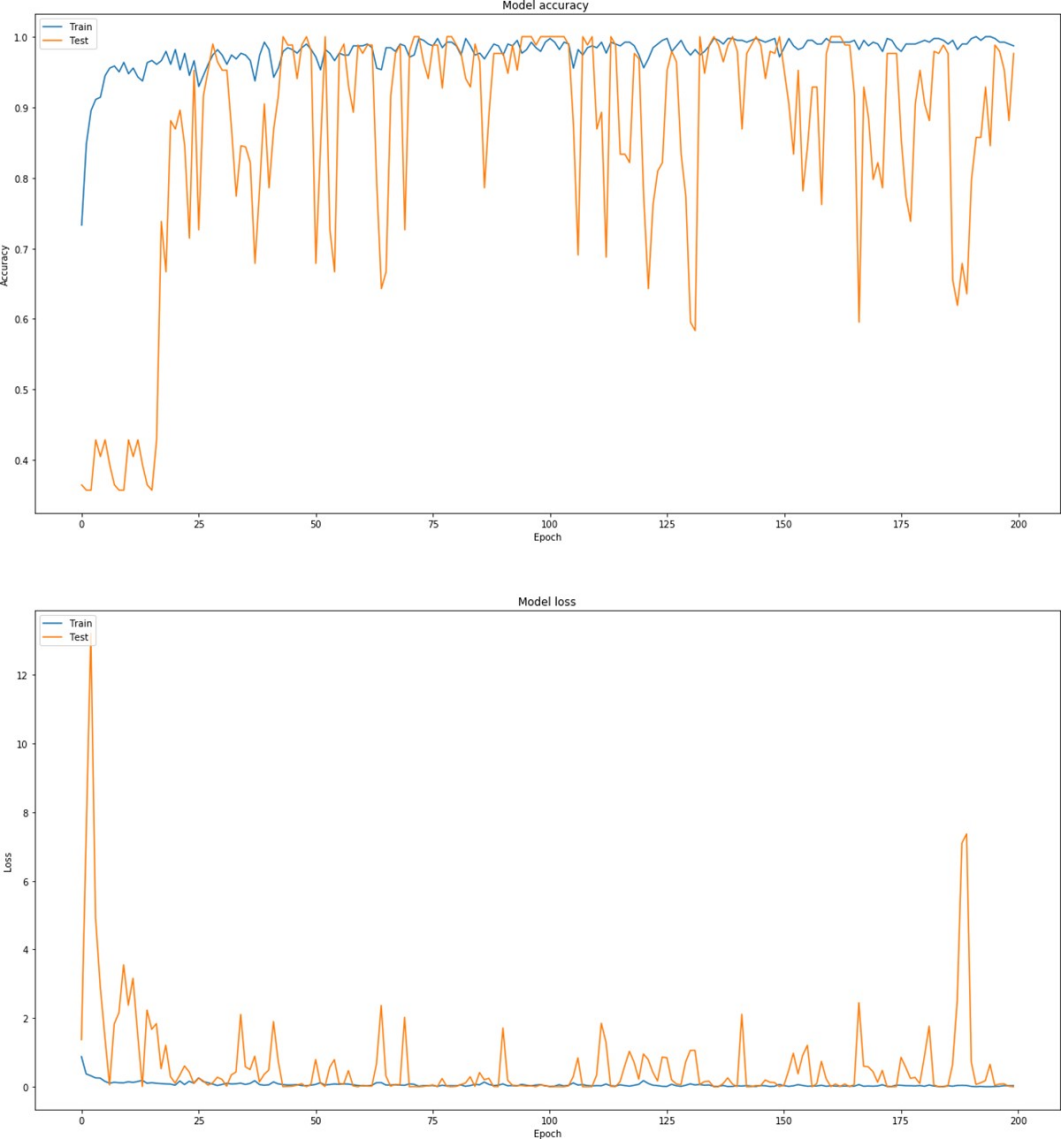
Precision curves for MobileNet (a) accuracy and (b) loss.

It is worth mentioning that we have conducted an exploratory analysis for all the benchmark CNNs, with different dropouts, while reducing the number of weights (to find a network complexity appropriate for the problem) to avoid overfitting [25]. Thus, we selected not only the best performing model of each CNN architecture concerning the accuracy, but at the same time, the best CNN which avoids overfitting in 10 runs. Indicative results of the five state-of-the-art CNN models for the selected optimum parameters, pixels, dropouts and batch sizes are gathered in S7 and S8 Tables. Furthermore, in S9 Table, the best confusion matrices for the five benchmark CNN models are gathered. Fig 9 illustrates the average prediction accuracy with the respective loss curve for the best performed state of the art CNN (MobileNet).

## 5. Discussion of results

The problem of diagnosis of bone metastasis in prostate cancer patients has been tackled with the use of CNN algorithms, which are considered applicable and powerful methods for detecting complex visual patterns in the field of medical image analysis. In the current work, a total of 586 images was acquired, that includes similar numbers of both healthy and malignant cases from metastatic prostate cancer patients. Several CNN architectures were tested, leading to the one that performed optimum under all hyperparameter selection cases and regularization methods, emerging classification accuracies ranging from 93.75% to 98.685%.

After a thorough exploration analysis, as reported in section 3, a CNN architecture with the following specifications: 3 convolutional layers, batch size = 16, dropout = 0.7 and dense nodes = 64, has provided the highest classification accuracy (97.38%) and the lowest loss (0.08) among all the architectures investigated in this work. Concerning the evaluation metrics for the proposed model configuration, as depicted in Table 4, it is observed that precision (0.969), recall (0.974), sensitivity (0.965), specificity (0.968) and F1 score (0.97) present the highest values when compared with those of the rest of the examined model configurations. It is worth mentioning that when it comes to the proposed architecture with a different number of dense nodes (32 or 128), it is proven that the produced classification model is still considerably accurate even though it provides a lower testing accuracy (97%) than that of the proposed CNN model.

To further discuss the results produced, we compare (even indirectly) the classification performance of our model with that of other ML algorithms and CNNs, which were reported in the literature and already applied in the particular problem of bone metastasis classification in nuclear medicine. Due to the fact that none of these research studies provide publicly available data, it is not feasible to accomplish a straightforward comparison with them. Thus, we gathered all the related research works in this domain and present them in Table 10 (as mentioned in the Introduction section), accompanied with the calculated classification accuracies and evaluation metrics, like sensitivity and specificity.

**Table 10:** Related works in bone scintigraphy imaging for prostate cancer metastasis using ML and CNN techniques.

	Reference	Year	ML Methods	Classification problem	Results
**Bone scintigraphy (bone scans)**	Sadik et al. [51]	2008	ANNs	Metastasis present or absent	Sensitivity 90% and accuracy 74%
	Wang et al. [58]	2016	DT and SVM	(2 classes) predict metastasis or not	87.58% ± 2.25%
	Dang J. (Master Thesis) [49]	2016	CNNs	(2 classes) metastatic / non-metastatic hotspots (*)	calculated accuracy of the testing set 0.89
	Belcher L. (Master Thesis) [50]	2017	CNNs	(2 classes) metastatic / non-metastatic hotspots	AUC score 0.937
	Horikoshi et al. [48]	2012	ANNs	metastatic disease or not	Sensitivity 90% and accuracy 83%
	Aslantas et al. [56]	2016	CADBOSS (ANNs)	(2 classes) Metastasis present or absent	The accuracy, sensitivity, and specificity were 92.30%, 94%, and 86.67%
**FDG PET or FDG PET-CT**	Bradshaw T/ et al. [59]	2018	CNNs	(2 classes) classification of benign and malignant bone lesions	0.88, 0.90, 0.85, and 0.90, concerning the accuracy, sensitivity, specificity & positive predictive value
	Furuya S. et al. [60]	2019	Deep Learning	(3 classes) 1) benign, 2) malignant, and 3) equivocal	Accuracies for benign, malignant and equivocal were 99.4%, 99.4% and 87.5%, respectively
	Furuya S. et al. [61]	2019	ResNet24	(3 classes) 1) benign in the head-and-neck region, 2) malignant in the head-and-neck region, and 3) equivocal in the head-and-neck region	Accuracies for 1), 2) and 3) were 97.3%, 97.8% and 96.2% respectively.
	Kawauchi K et al. [62]	2019	CNN	(2 classes) classifying these patients by sex (male-female)	The accuracy values were 95.9% for male and 90.2% for female.
	Kawauchi K et al. [63]	2018	CNNs	(2 classes) predict whether physician’s further diagnosis is required or not	Accuracy 93.2±3.9%

* Only the hotspots found in the spine had been used to train the CNN.

A comparative analysis has been further conducted by authors to demonstrate the novelty of the proposed method. This analysis concerns recent ML and CNN techniques presented in related works in bone scintigraphy imaging, along with their specifications, as listed in Table 10. As regards the previous works of [49] and [50] in bone scintigraphy classification, they employ CNNs for metastatic / non-metastatic hotspots classification. They are highly related to this research study and provide classification accuracies up to 89%. Taking into consideration only the CNN methods with 2 classes (the same number of classes as the proposed model), then the highest possible accuracy value (95.9%) is achieved in [62]. Overall, comparing the classification accuracy value of the proposed CNN method (97.38%) with those of the reported methods listed in Table 10, authors come to the conclusion that the proposed CNN approach outperforms all the previous ML and deep learning techniques in bone scintigraphy imaging.

Besides accuracy, several other evaluation metrics like precision, recall, sensitivity, specificity and F1 score, as listed in Tables 7, 8 and 9, were used by authors to measure the classification performance of the proposed model as well as other state-of-the-art CNNs, commonly used for image classification problems. In regard to the testing accuracy values for the specific problem, the suggested algorithm seems to outperform all the reported benchmark CNNs (their testing accuracy is up to 96.7%), except from MobileNet, whose accuracy results (98.13%) are slightly higher than that of the proposed approach. The produced values for the rest of the reported metrics of all the examined architectures present a similar picture, thus, concluding that our model exhibits better or similar performance to the other state-of-the-art CNN architectures. This study validates the premise that CNNs are algorithms that can offer high accuracy in medical image classification-based problems. This has a direct application on medical imaging where the automatic identification of diseases is crucial for the patients. The main outcomes of this study can be summarized as follows:

- The proposed CNN method exhibits outstanding performance when RGB analysis is performed for the examined images of this case study. This can be emanated from the fact that the results produced after the application of CNN architecture are based on distinct features, that appear specifically on the bone metastasis presence scans, compared to the healthy scans (no malignant spots).
- The proposed CNN architecture is superior to four out of five benchmark and well-known CNN architectures (ResNet50, VGG16, Inception V3 and Xception), which have been efficiently used in medical image processing problems. As it is observed from Tables 8, 9 and 10, the results deriving from the application of the CNN method, are better in terms of classification accuracy, prediction, sensitivity and F1 score, than those coming from popular and well-known CNN approaches, found in the relevant literature. In the case of MobileNet, it shows a relatively similar classification accuracy to the proposed model.
- The proposed CNN models appear to have significant potential since they exhibit better performance with less running time and simpler architecture than other well-known CNN architectures, considering the case of bone metastasis classification of whole-body scans.
- The proposed bone scan CNN performs efficiently, despite the fact that it was trained on a small number of images.
- Overall, for the purpose of medical image analysis and classification, the CNN methodology is proven to be powerful enough in the nuclear medicine domain and particularly, for bone scintigraphy, outweighing the popular CNN architectures for image analysis, like VGG16, ResNet50, MobileNet, InceptionV3 and Xception.

## 6. Conclusions and future work

It is evident that medical imaging paired with machine learning techniques, specifically with CNNs, is considerably valuable for clinical diagnosis of bone metastasis. Considering and exploiting all new enhancements in the field of deep learning, a simpler, faster and more accurate set of CNN networks for classification is proposed. In the case where only bone scan images are used at the input level, the classifier can identify the presence of bone metastasis in prostate cancer patients. The proposed CNN-based method in this work, outperforms all other popular and previously proposed CNN methods, achieving excellent performance in terms of classification accuracy, precision, recall, sensitivity and specificity indicators. Accordingly, the effectiveness of the suggested improvements to the network architecture and hyperparameter configuration are presented in this research study. The experimental results demonstrate that the proposed method achieves outstanding performance comparing with other methods that make use of convolutional neural networks. In particular, this approach allows an easier, faster and more precise interpretation of scintigraphy images, which can have a positive impact on diagnosis accuracy as well as on decision making, regarding the treatment that will be further administered.

Even though this CNN approach uses a relatively small dataset of patients, this work suggests that bone scintigraphy, combined with the CNN trained models, can have a considerable effect in the detection of bone metastasis. In our next step, more images will be gathered regarding patients suffering from prostate cancer, as well as patients suffering from other types of metastatic cancer, like breast, kidney, lung or thyroid cancer in order to thoroughly investigate the proposed architecture. The results seem quite promising and really encouraging when computer aided diagnosis is concerned, making the proposed network more useful for clinical routine work.

Summarizing, future plans include further investigation of both deep learning methods and the proposed architecture in two directions; to collect more bone scans from patients suffering from various types of metastatic cancer (except prostate cancer) and to examine interpretability of the model and workflow integration.

## Supporting information

S1 Appendix Convolutional neural networks basics. (DOCX)

S2 Appendix Classification performance metrics. (DOCX)

S1 Table Performance metrics for the best CNN network for 10 runs (batch size = 16, dropout = 0.7). (DOCX)

S2 Table CNN model 1 (epochs = 200, dropout = 0.2, pixel = 256x256x3), and dense nodes = 64. (DOCX)

S3 Table Model 1 (epochs = 200, dropout = 0.5, pixel = 256x256x3) dense nodes = 64. (DOCX)

S4 Table CNN Model 1 (epochs = 200, dropout = 0.9, pixel = 256x256x3) dense nodes = 64. (DOCX)

S5 Table CNN model (epochs = 200, dropout = 0.7, pixel = 300x300x3) runs for different batch sizes. (DOCX)

S6 Table CNN model with 4 conv (8,16,32,64), epochs = 200, dropout = 0.7, pixel = 256 x 256 x 3, different dense nodes and batch size = 16. (DOCX)

S7 Table 10 Runs for VGG16 (epochs = 200, dropout = 0.7, pixel = 256 × 256x3), resNet50 (epochs = 200, dropout = 0.2, pixel = 300 × 300 x 3) and MobileNet (epochs = 200, dropout = 0.2, pixel = 300 × 300 x 3). (DOCX)

S8 Table 10 Runs for inceptionV3 (epochs = 200, dropout = 0.7, pixel = 250 × 250 x 3, dense = 2x1500) and xception (epochs = 200, dropout = 0.7, pixel = 300 × 300 x 3, dense = 2x512). (DOCX)

S9 Table Confusion matrices for state of the art CNN models. (DOCX)
