# Supplementary material for: Bone metastasis classification using whole body images from prostate cancer patients based on convolutional neural networks application
Source: PLoS One. 2020 Aug 14;15(8):e0237213. doi: 10.1371/journal.pone.0237213 (PMC7428190; doi:10.1371/journal.pone.0237213)
Supplement: S1 Appendix — (DOCX) [file pone.0237213.s001.docx]

**S1 Appendix. Convolutional Neural Networks Basics**

A convolutional neural network is a type of artificial neural network that uses connections between the layers to maintain the spatial relationships within the data. It is structured with layers that perform convolutions, followed by layers that perform pooling, several times over. Gradient descent is used for optimization and backpropagation for updating the weights after an iteration [22]. The final outputs are computed by fully connected layers, located at the end of the network.

The main elements/components of a CNN architecture are: (i) Convolutional layers, (ii) Activation Layer, (iii) Pooling [79], (iv) Dropout regularization [80] and (v) Batch normalization [81]. A typical convolutional neural network consists of one or more filters (i.e., convolutional layers), followed by fully connected layers and an activation function, like a standard ANN [72].

The input matrix is analyzed by a fixed number of filters that run through the input with a constant step size. Each filter moves from the left to the right and jumps to the next lower line after each pass. The padding function can be used to define how the filter should behave when it reaches the end of the line. Each application of a filter creates a new input (i.e., new matrix) that is the result of a convolution [82], [22]. The activation functions are generally the very simple rectified linear units, or ReLUs [83].

Afterwards, the pooling layer (or down-sampling layer) [72] is used. This layer aggregates the results of the convolutional layers. Pooling serves to pass only the most relevant signals (e.g., the highest values) to the next layers to achieve a more abstract representation of the content and to reduce the number of parameters of a layer [22], [82].

The sequence of convolutional layers followed by a pooling layer can be used as often as necessary. This decision depends on the use case, the case context and the available data. After the sequence of convolutional and pooling layers, the last pooling layer is followed by one or several fully connected layers. These layers converge the aggregated results into a feature vector, which is then used for classification. This means that the created feature vector is compared with existing trained feature vectors and assigned to a specific class. This, in turn, outputs the appropriate result [22].

**References**

79. Springenberg JT, Dosovitskiy A, Brox T, Riedmiller M. Striving for simplicity: The all convolutional net. In: 3rd International Conference on Learning Representations, ICLR 2015 - Workshop Track Proceedings. 2015.

80. Srivastava N, Hinton G, Krizhevsky A, Sutskever I, Salakhutdinov R. Dropout: A simple way to prevent neural networks from overfitting. Journal of Machine Learning Research. 2014;15:1929–58.

81. Ioffe S, Szegedy C. Batch normalization: Accelerating deep network training by reducing internal covariate shift. In: 32nd International Conference on Machine Learning, ICML 2015. 2015.

82. Lin M, Chen Q, Yan S. Network in network. In: 2nd International Conference on Learning Representations, ICLR 2014 - Conference Track Proceedings. 2014.

83. Clevert DA, Unterthiner T, Hochreiter S. Fast and accurate deep network learning by exponential linear units (ELUs). In: 4th International Conference on Learning Representations, ICLR 2016 - Conference Track Proceedings. 2016.
