# Supplementary material for: Bone metastasis classification using whole body images from prostate cancer patients based on convolutional neural networks application
Source: PLoS One. 2020 Aug 14;15(8):e0237213. doi: 10.1371/journal.pone.0237213 (PMC7428190; doi:10.1371/journal.pone.0237213)
Supplement: S2 Appendix — (DOCX) [file pone.0237213.s002.docx]

The mathematical formulations of the six well-known performance metrics are as follows:

| $Accuracy=\frac{TP+TN}{TP+TN+FP+FN}$ | (1) |
| --- | --- |
| $Precision=\frac{TP}{TP+FP}$ | (2) |
| $Recall=\frac{TP}{TP+TN}$ | (3) |
| $F1-score=\frac{2*(Recall\times Precision)}{Recall+Precision}$ | (4) |
| $Sensitivity=\frac{TP}{TP+FN}$ | (5) |
| $Specificity=\frac{TN}{TN+FP}$ | (6) |

Accuracy is the ratio of correctly predicted observations. Precision refers to the ratio of the number of category samples correctly predicted to the total number of samples, all predicted for that category. Recall is the ratio of the number of samples correctly predicted for the class to the total number of samples for the class. It is also called sensitivity or hit rate. F1 score is the harmonic mean of precision and sensitivity of the classification. The larger these performance values are, the better the performance of a method is. Sensitivity is the proportion of images which test positive for the malignancy (bone metastasis) among those which have the metastasis/malignancy. Specificity is an evaluation metric, that measures the balance between the corrected negative images and the sum of numerator and wrong labeled positive images.
