# Supplementary material for: Bone metastasis classification using whole body images from prostate cancer patients based on convolutional neural networks application
Source: PLoS One. 2020 Aug 14;15(8):e0237213. doi: 10.1371/journal.pone.0237213 (PMC7428190; doi:10.1371/journal.pone.0237213)
Supplement: S1 Table — (DOCX) [file pone.0237213.s003.docx]

**S1 Table.** Performance metrics for the best CNN network for 10 runs (batch size = 16, dropout = 0.7).

| **Runs** |  | **Precision** | **Recall** | **F1-Score** | **Sensitivity** | **Specificity** |
| --- | --- | --- | --- | --- | --- | --- |
| Run 1 | Malignant | 1 | 0,97 | 0,98 | 1,00 | 0,94 |
|  | Benign | 0,94 | 1 | 0,97 | 0,94 | 1,00 |
| Run 2 | Malignant | 0,98 | 0,97 | 0,98 | 0,98 | 0,92 |
|  | Benign | 0,92 | 0,96 | 0,94 | 0,92 | 0,98 |
| Run 3 | Malignant | 1 | 0,96 | 0,98 | 1,00 | 0,94 |
|  | Benign | 0,94 | 1 | 0,97 | 0,94 | 1,00 |
| Run 4 | Malignant | 0,98 | 1 | 0,99 | 0,98 | 1,00 |
|  | Benign | 1 | 0,96 | 0,98 | 1,00 | 0,98 |
| Run 5 | Malignant | 1 | 0,96 | 0,98 | 1,00 | 0,94 |
|  | Benign | 0,94 | 1 | 0,97 | 0,94 | 1,00 |
| Run 6 | Malignant | 0,98 | 0,97 | 0,98 | 0,98 | 0,93 |
|  | Benign | 0,93 | 0,96 | 0,94 | 0,92 | 0,98 |
| Run 7 | Malignant | 1 | 0,95 | 0,97 | 1,00 | 0,91 |
|  | Benign | 0,91 | 1 | 0,95 | 0,91 | 1,00 |
| Run 8 | Malignant | 0,98 | 0,98 | 0,98 | 0,98 | 0,97 |
|  | Benign | 0,97 | 0,97 | 0,97 | 0,97 | 0,98 |
| Run 9 | Malignant | 0,98 | 0,97 | 0,98 | 0,94 | 0,95 |
|  | Benign | 0,92 | 0,96 | 0,94 | 0,92 | 0,98 |
| Run 10 | Malignant | 0,97 | 1 | 0,98 | 0,97 | 1,00 |
|  | Benign | 1 | 0,94 | 0,97 | 1,00 | 0,97 |
| **Average** | Malignant | **0,987** | **0,973** | **0,980** | **0,984** | **0,948** |
|  | Benign | **0,947** | **0,975** | **0,960** | **0,945** | **0,987** |
