# Supplementary material for: Bone metastasis classification using whole body images from prostate cancer patients based on convolutional neural networks application
Source: PLoS One. 2020 Aug 14;15(8):e0237213. doi: 10.1371/journal.pone.0237213 (PMC7428190; doi:10.1371/journal.pone.0237213)
Supplement: S2 Table — (DOCX) [file pone.0237213.s004.docx]

**S2 Table**. CNN Model 1 (epochs=200, dropout=0.2, pixel=256x256x3), and dense nodes=64.

|  | Batch size=8 | | | | Batch size=16 | | | | Batch size=32 | | | | Batch size=64 | | | |
| --- | --- | --- | --- | --- | --- | --- | --- | --- | --- | --- | --- | --- | --- | --- | --- | --- |
|  | Acc. Val | Loss Val | Acc Test | Loss Test | Acc. Val | Loss Val | Acc Test | Loss Test | Acc. Val | Loss Val | Acc Test | Loss Test | Acc. Val | Loss Val | Acc Test | Loss Test |
| Run1 | 95,83 | 0,10 | 94,31 | 0,14 | 93,75 | 0,13 | 96,25 | 0,10 | 94,79 | 0,12 | 90,63 | 0,32 | 85,93 | 0,68 | 85,93 | 0,47 |
| Run2 | 93,75 | 0,13 | 93,18 | 0,18 | 90,63 | 0,20 | 97,50 | 0,09 | 95,83 | 0,14 | 90,63 | 0,23 | 92,18 | 0,24 | 92,18 | 0,38 |
| Run3 | 96,88 | 0,10 | 92,04 | 0,22 | 94,79 | 0,11 | 92,50 | 0,16 | 94,79 | 0,16 | 100 | 0,08 | 95,31 | 0,18 | 92,18 | 0,30 |
| Run4 | 96,88 | 0,07 | 98,86 | 0,07 | 97,92 | 0,10 | 93,75 | 0,24 | 97,91 | 0,06 | 92,19 | 0,19 | 95,31 | 0,10 | 95,31 | 0,23 |
| Run5 | 94,79 | 0,28 | 93,18 | 0,25 | 94,79 | 0,15 | 97,50 | 0,08 | 90,63 | 0,21 | 90,63 | 0,27 | 93,75 | 0,15 | 96,88 | 0,07 |
| **AVE** | 95,62 | 0,14 | **94,31** | 0,17 | 94,38 | 0,14 | **95,50** | 0,13 | 94,79 | 0,14 | 92,81 | 0,22 | 92,50 | 0,27 | 92,50 | 0,29 |
