# Supplementary material for: Bone metastasis classification using whole body images from prostate cancer patients based on convolutional neural networks application
Source: PLoS One. 2020 Aug 14;15(8):e0237213. doi: 10.1371/journal.pone.0237213 (PMC7428190; doi:10.1371/journal.pone.0237213)
Supplement: S3 Table — (DOCX) [file pone.0237213.s005.docx]

**S3 Table**. Model 1 (epochs=200, dropout=0.5, pixel=256x256x3) dense nodes=64.

|  | Batch size=8 | | | | Batch size=16 | | | | Batch size=32 | | | | Batch size=64 | | | |
| --- | --- | --- | --- | --- | --- | --- | --- | --- | --- | --- | --- | --- | --- | --- | --- | --- |
|  | Acc. Val | Loss Val | Acc Test | Loss Test | Acc. Val | Loss Val | Acc Test | Loss Test | Acc. Val | Loss Val | Acc Test | Loss Test | Acc. Val | Loss Val | Acc Test | Loss Test |
| Run1 | 93,75 | 0,22 | 94,31 | 0,21 | 96,88 | 0,11 | 96,25 | 0,10 | 88,84 | 0,22 | 87,50 | 0,34 | 95,31 | 0,21 | 93,75 | 0,20 |
| Run2 | 94,79 | 0,11 | 88,63 | 0,32 | 95,83 | 0,08 | 96,25 | 0,11 | 98,95 | 0,03 | 93,75 | 0,18 | 92,18 | 0,12 | 95,31 | 0,11 |
| Run3 | 95,83 | 0,13 | 95,45 | 0,17 | 95,83 | 0,09 | 95,00 | 0,11 | 94,79 | 0,15 | 93,75 | 0,12 | 92,18 | 0,15 | 93,75 | 0,03 |
| Run4 | 96,88 | 0,15 | 93,18 | 0,12 | 94,79 | 0,17 | 93,75 | 0,19 | 92,70 | 0,21 | 90,63 | 0,22 | 92,15 | 0,14 | 93,75 | 0,04 |
| Run5 | 92,71 | 0,22 | 97,72 | 0,07 | 95,83 | 0,10 | 97,50 | 0,08 | 96,88 | 0,10 | 95,31 | 0,24 | 95,31 | 0,06 | 93,75 | 0,15 |
| **AVE** | 94,79 | 0,17 | 93,86 | 0,18 | 95,83 | 0,11 | **95,75** | **0,12** | 94,43 | 0,14 | 92,19 | 0,22 | 93,43 | 0,14 | **94,06** | **0,07** |
