# Supplementary material for: Bone metastasis classification using whole body images from prostate cancer patients based on convolutional neural networks application
Source: PLoS One. 2020 Aug 14;15(8):e0237213. doi: 10.1371/journal.pone.0237213 (PMC7428190; doi:10.1371/journal.pone.0237213)
Supplement: S4 Table — (DOCX) [file pone.0237213.s006.docx]

**S4 Table**. CNN Model 1 (epochs=200, dropout=0.9, pixel=256x256x3) dense nodes=64.

|  | Batch size=8 | | | | Batch size=16 | | | | Batch size=32 | | | | | Batch size=64 | | | |
| --- | --- | --- | --- | --- | --- | --- | --- | --- | --- | --- | --- | --- | --- | --- | --- | --- | --- |
|  | Acc. Val | Loss Val | Acc Test | Loss Test | Acc. Val | Loss Val | Acc Test | Loss Test | Acc. Val | Loss Val | Acc Test | Loss Test | Acc. Val | | Loss Val | Acc Test | Loss Test |
| Run1 | 95,83 | 0,13 | 97,72 | 0,14 | 95,83 | 0,13 | 93,75 | 0,17 | 95,83 | 0,16 | 93,75 | 0,18 | 93,75 | | 0,22 | 92,18 | 0,20 |
| Run2 | 95,83 | 0.14 | 95,45 | 0.15 | 92,71 | 0,21 | 85,00 | 0,25 | 93,75 | 0,19 | 96,87 | 0,16 | 78,13 | | 0,38 | 81,25 | 0,36 |
| Run3 | 95,83 | 0,10 | 96,59 | 0,15 | 96,88 | 0,15 | 92,50 | 0,18 | 85,42 | 0,31 | 89,10 | 0,32 | 96,88 | | 0,24 | 90,63 | 0,29 |
| Run4 | 97,92 | 0.12 | 95,45 | 0.16 | 97,92 | 0,18 | 97,50 | 0,17 | 92,71 | 0,24 | 87,50 | 0,32 | 90,63 | | 0,32 | 85,93 | 0,35 |
| Run5 | 94,79 | 0,18 | 95,45 | 0,14 | 95,83 | 0,17 | 90,00 | 0,24 | 92,71 | 0,24 | 87,50 | 0,32 | 89,06 | | 0,31 | 84,38 | 0,35 |
| **AVE** | *96,04* | *0,08* | ***96,13*** | ***0,09*** | *95,83* | *0,17* | ***91,75*** | ***0,20*** | *92,08* | *0,23* | ***90,94*** | ***0,26*** | 89,69 | | 0,30 | **86,87** | **0,31** |
