# Supplementary material for: Bone metastasis classification using whole body images from prostate cancer patients based on convolutional neural networks application
Source: PLoS One. 2020 Aug 14;15(8):e0237213. doi: 10.1371/journal.pone.0237213 (PMC7428190; doi:10.1371/journal.pone.0237213)
Supplement: S5 Table — (DOCX) [file pone.0237213.s007.docx]

**S5 Table**. CNN Model (epochs=200, dropout=0.7, pixel=300x300x3) runs for different batch sizes.

|  | Batch size=8 | | | | Batch size=16 | | | | Batch size=32 | | | | | Batch size=64 | | | |
| --- | --- | --- | --- | --- | --- | --- | --- | --- | --- | --- | --- | --- | --- | --- | --- | --- | --- |
|  | Acc. Val | Loss Val | Acc Test | Loss Test | Acc. Val | Loss Val | Acc Test | Loss Test | Acc. Val | Loss Val | Acc Test | Loss Test | Acc. Val | | Loss Val | Acc Test | Loss Test |
| Run1 | 94,79 | 0,11 | 97,72 | 0,09 | 95,83 | 0,09 | 92,50 | 0,24 | 97,92 | 0,09 | 98,44 | 0,07 | 100 | | 0,03 | 96,87 | 0,11 |
| Run2 | 94,79 | 0,39 | 93,18 | 0,19 | 96,88 | 0,09 | 96,25 | 0,09 | 98,96 | 0,05 | 92,19 | 0,19 | 100 | | 0,07 | 96,87 | 0,17 |
| Run3 | 94,79 | 0,13 | 96,59 | 0,07 | 92,71 | 0,11 | 98,75 | 0,07 | 92,71 | 0,26 | 90,62 | 0,30 | 100 | | 0,09 | 96,87 | 0,11 |
| Run4 | 95,83 | 0,10 | 96,59 | 0,14 | 96,88 | 0,55 | 95,00 | 0,17 | 93,75 | 0,20 | 90,62 | 0,34 | 100 | | 0,09 | 98,44 | 0,06 |
| Run5 | 96,87 | 0,05 | 97,72 | 0,07 | 98,96 | 0,09 | 98,75 | 0,06 | 94,79 | 0,13 | 98,44 | 0,07 | 100 | | 0,10 | 98,44 | 0,10 |
| **AVE** | 95,41 | 0,16 | **96,36** | 0,11 | 96,25 | 0,19 | **96,25** | 0,13 | 95,63 | 0,15 | **94,06** | 0,19 | **100** | | **0,08** | **97,50** | **0,11** |
