# Supplementary material for: Bone metastasis classification using whole body images from prostate cancer patients based on convolutional neural networks application
Source: PLoS One. 2020 Aug 14;15(8):e0237213. doi: 10.1371/journal.pone.0237213 (PMC7428190; doi:10.1371/journal.pone.0237213)
Supplement: S6 Table — (DOCX) [file pone.0237213.s008.docx]

**S8 Table**. CNN Model with 4 conv (8,16,32,64), epochs=200, dropout=0.7, pixel=256x256x3, different dense nodes and batch size=16

|  | **Dense nodes=32** | | | | **Dense nodes=64** | | | | **Dense nodes=128** | | | |
| --- | --- | --- | --- | --- | --- | --- | --- | --- | --- | --- | --- | --- |
|  | Acc. Val | Loss Val | Acc Test | Loss Test | Acc. Val | Loss Val | Acc Test | Loss Test | Acc. Val | Loss Val | Acc Test | Loss Test |
| Run1 | 96,88 | 0,10 | 97,50 | 0,12 | 95,83 | 0,16 | 97,50 | 0,09 | 94,79 | 0,107 | 96,25 | 0,122 |
| Run2 | 97,91 | 0,07 | 97,50 | 0,08 | 97,92 | 0,11 | 98,75 | 0,06 | 97,91 | 0,103 | 96,25 | 0,078 |
| Run3 | 95,83 | 0,14 | 97,50 | 0,06 | 97,91 | 0,10 | 95,00 | 0,12 | 95,83 | 0,131 | 92,5 | 0,174 |
| Run4 | 96,88 | 0,09 | 97,50 | 0,11 | 95,83 | 0,09 | 93,75 | 0,18 | 94,79 | 0,106 | 100 | 0,07 |
| Run5 | 94,79 | 0,11 | 96,25 | 0,10 | 98,95 | 0,07 | 96,25 | 0,10 | 87,5 | 0,285 | 83,75 | 0,35 |
| Average | **96,46** | **0,10** | **97,25** | **0,09** | 97,29 | 0,10 | **96,25** | **0,11** | 94,16 | 0,15 | **93,75** | **0,16** |
