# Supplementary material for: Bone metastasis classification using whole body images from prostate cancer patients based on convolutional neural networks application
Source: PLoS One. 2020 Aug 14;15(8):e0237213. doi: 10.1371/journal.pone.0237213 (PMC7428190; doi:10.1371/journal.pone.0237213)
Supplement: S7 Table — (DOCX) [file pone.0237213.s009.docx]

**S7 Table**. **10 Runs for** VGG16 (epochs = 200, dropout = 0.7, pixel = 256 × 256x3), ResNet50 (epochs = 200, dropout = 0.2, pixel = 300 × 300 x 3) and MobileNet (epochs = 200, dropout = 0.2, pixel = 300 × 300 x 3)

|  | **VGG16 (batch size=16)** | | | | **ResNet50 (batch size=8)** | | | | **MobileNet (batch size=16)** | | | |
| --- | --- | --- | --- | --- | --- | --- | --- | --- | --- | --- | --- | --- |
|  | Val | Loss | Test | T.Loss | Val | Loss | Test | T.Loss | Val | Loss | Test | T.Loss |
| Run 1 | 93,75 | 0,13 | 100,00 | 0,06 | 93,75 | 0,13 | 98,86 | 0,03 | 92,70 | 0,21 | 98,75 | 0,02 |
| Run 2 | 98,43 | 0,08 | 93,75 | 0,13 | 92,71 | 0,14 | 94,32 | 0,13 | 98,96 | 0,03 | 97,50 | 0,12 |
| Run 3 | 96,88 | 0,14 | 95,31 | 0,17 | 93,75 | 0,13 | 94,32 | 0,24 | 96,88 | 0,07 | 98,75 | 0,02 |
| Run 4 | 90,63 | 0,23 | 96,88 | 0,12 | 94,79 | 0,11 | 98,86 | 0,02 | 95,83 | 0,10 | 98,75 | 0,02 |
| Run 5 | 95,31 | 0,14 | 95,31 | 0,13 | 92,71 | 0,15 | 95,45 | 0,08 | 98,95 | 0,01 | 98,75 | 0,02 |
| Run 6 | 98,43 | 0,09 | 96,88 | 0,22 | 96,87 | 0,05 | 97,72 | 0,08 | 96,88 | 0,03 | 98,75 | 0,05 |
| Run 7 | 93,75 | 0,15 | 87,50 | 0,28 | 97,92 | 0,07 | 88,64 | 0,29 | 97,91 | 0,04 | 100,00 | 0,03 |
| Run 8 | 85,94 | 0,29 | 87,50 | 0,27 | 97,92 | 0,04 | 98,63 | 0,02 | 97,91 | 0,04 | 97,50 | 0,06 |
| Run 9 | 92,19 | 0,18 | 93,75 | 0,16 | 92,71 | 0,19 | 86,36 | 0,53 | 100,00 | 0,01 | 96,25 | 0,12 |
| Run 10 | 89,10 | 0,25 | 90,62 | 0,17 | 96,87 | 0,15 | 96,59 | 0,14 | 96,88 | 0,16 | 96,25 | 0,14 |
| Average | 93,44 | 0,17 | 93,75 | 0,17 | 95,00 | 0,12 | 94,98 | 0,16 | 97,29 | 0,07 | 98,13 | 0,06 |
