# Supplementary material for: Bone metastasis classification using whole body images from prostate cancer patients based on convolutional neural networks application
Source: PLoS One. 2020 Aug 14;15(8):e0237213. doi: 10.1371/journal.pone.0237213 (PMC7428190; doi:10.1371/journal.pone.0237213)
Supplement: S8 Table — (DOCX) [file pone.0237213.s010.docx]

**S8 Table**. 10 Runs for InceptionV3 (epochs = 200, dropout = 0.7, pixel = 250 × 250 x 3, dense=2x1500) and Xception (epochs = 200, dropout = 0.7, pixel = 300 × 300 x 3, dense=2x512)

|  | **InceptionV3 (batch size=16)** | | | | **Xception (batch size=8)** | | | |
| --- | --- | --- | --- | --- | --- | --- | --- | --- |
|  | Val | Loss | Test | T.Loss | Val | Loss | Test | T.Loss |
| Run 1 | 97,90 | 0,09 | 98,75 | 0,06 | 98,95 | 0,05 | 96,59 | 0,21 |
| Run 2 | 92,70 | 0,13 | 97,50 | 0,06 | 96,87 | 0,11 | 98,86 | 0,11 |
| Run 3 | 98,96 | 0,08 | 98,75 | 0,03 | 100,00 | 0,01 | 97,72 | 0,09 |
| Run 4 | 93,75 | 0,12 | 97,50 | 0,07 | 98,96 | 0,06 | 97,72 | 0,13 |
| Run 5 | 95,83 | 0,23 | 96,25 | 0,43 | 92,71 | 0,26 | 94,32 | 0,12 |
| Run 6 | 97,92 | 0,07 | 95,00 | 0,09 | 98,96 | 0,06 | 95,45 | 0,10 |
| Run 7 | 98,96 | 0,04 | 96,25 | 0,07 | 93,75 | 0,12 | 87,50 | 0,43 |
| Run 8 | 92,71 | 0,13 | 97,50 | 0,08 | 97,92 | 0,03 | 97,72 | 0,04 |
| Run 9 | 93,75 | 0,20 | 92,50 | 0,18 | 97,92 | 0,06 | 98,86 | 0,05 |
| Run 10 | 92,71 | 0,12 | 91,25 | 0,28 | 98,96 | 0,03 | 98,86 | 0,03 |
| Average | 95,52 | 0,12 | 96,13 | 0,14 | 97,50 | 0,08 | 96,36 | 0,13 |
