# Supplementary material for: Bone metastasis classification using whole body images from prostate cancer patients based on convolutional neural networks application
Source: PLoS One. 2020 Aug 14;15(8):e0237213. doi: 10.1371/journal.pone.0237213 (PMC7428190; doi:10.1371/journal.pone.0237213)
Supplement: S9 Table — (DOCX) [file pone.0237213.s011.docx]

**S11 Table**. Confusion matrices for state of the art CNN models

| \| ***Confusion*** \| malignant \| healthy \| \| --- \| --- \| --- \| \| malignant \| 58 \| 4 \| \| healthy \| 3 \| 23 \|  1. VGG16 | \| ***Confusion*** \| malignant \| healthy \| \| --- \| --- \| --- \| \| malignant \| 63 \| 1 \| \| healthy \| 0 \| 24 \|  1. ResNet50 |
| --- | --- | --- | --- | --- | --- | --- | --- | --- | --- | --- | --- | --- | --- | --- | --- | --- | --- | --- | --- |
| \| ***Confusion*** \| malignant \| healthy \| \| --- \| --- \| --- \| \| malignant \| 58 \| 1 \| \| healthy \| 0 \| 29 \|  1. MobileNet | \| ***Confusion*** \| malignant \| healthy \| \| --- \| --- \| --- \| \| malignant \| 52 \| 0 \| \| healthy \| 2 \| 34 \|  1. Inception V3 |
| \| ***Confusion*** \| malignant \| healthy \| \| --- \| --- \| --- \| \| malignant \| 56 \| 0 \| \| healthy \| 1 \| 31 \|  1. Xception |  |
